# Supplementary material for: Modelling TFE renal cell carcinoma in mice reveals a critical role of WNT signaling
Source: eLife. 2016 Sep 26;5:e17047. doi: 10.7554/eLife.17047 (PMC5036965; doi:10.7554/eLife.17047)
Supplement: Figure 3—source data 1. — The genes are ranked by decreasing signed ratio (KSP_P0/CTL). DOI: http://dx.doi.org/10.7554/eLife.17047.008 [file elife-17047-fig3-data1.docx]

|  | | | |
| --- | --- | --- | --- |
| **Probe Set ID** | **Gene Symbol** | **Gene Title** | **ratio (KSP_P0/CTL)** |
| 1427262_at | Xist | inactive X specific transcripts | 410,2409275 |
| 1436936_s_at | Xist | inactive X specific transcripts | 151,7494387 |
| 1427263_at | Xist | inactive X specific transcripts | 41,58654297 |
| 1448303_at | Gpnmb | glycoprotein (transmembrane) nmb | 10,61358979 |
| 1418866_at | Cyp24a1 | cytochrome P450, family 24, subfamily a, polypeptide 1 | 4,937836094 |
| 1449151_at | Cdk18 | cyclin-dependent kinase 18 | 4,316468334 |
| 1416361_a_at | Dync1i1 | dynein cytoplasmic 1 intermediate chain 1 | 4,090833372 |
| 1450344_a_at | Ptger3 | prostaglandin E receptor 3 (subtype EP3) | 3,848013046 |
| 1416325_at | Crisp1 | cysteine-rich secretory protein 1 | 3,834705694 |
| 1449475_at | Atp12a | ATPase, H+/K+ transporting, nongastric, alpha polypeptide | 3,809611727 |
| 1419665_a_at | Nupr1 | nuclear protein 1 | 3,780990079 |
| 1418283_at | Cldn4 | claudin 4 | 3,420274195 |
| 1419666_x_at | Nupr1 | nuclear protein 1 | 3,31193314 |
| 1449133_at | Sprr1a | small proline-rich protein 1A | 3,163297279 |
| 1417089_a_at | Ckmt1 | creatine kinase, mitochondrial 1, ubiquitous | 3,091564393 |
| 1455531_at | Mfsd4 | major facilitator superfamily domain containing 4 | 3,085221266 |
| 1448502_at | Slc16a7 | solute carrier family 16 (monocarboxylic acid transporters), member 7 | 3,052132975 |
| 1439200_x_at | --- | --- | 3,037316262 |
| 1448998_at | Lpo | lactoperoxidase | 2,9532474 |
| 1436021_at | Mfsd4 | major facilitator superfamily domain containing 4 | 2,919398785 |
| 1449195_s_at | Cxcl16 | chemokine (C-X-C motif) ligand 16 | 2,918876107 |
| 1422612_at | Hk2 | hexokinase 2 | 2,900931546 |
| 1416460_at | Miox | myo-inositol oxygenase | 2,765569504 |
| 1418949_at | Gdf15 | growth differentiation factor 15 | 2,750952798 |
| 1456395_at | Ppargc1a | peroxisome proliferative activated receptor, gamma, coactivator 1 alpha | 2,713649997 |
| 1449166_at | S100a14 | S100 calcium binding protein A14 | 2,706462274 |
| 1437751_at | Ppargc1a | peroxisome proliferative activated receptor, gamma, coactivator 1 alpha | 2,698106944 |
| 1417531_at | Cyp2j5 | cytochrome P450, family 2, subfamily j, polypeptide 5 | 2,680061172 |
| 1422139_at | Plau | plasminogen activator, urokinase | 2,672368324 |
| 1448954_at | Nrip3 | nuclear receptor interacting protein 3 | 2,654596439 |
| 1432418_a_at | Ckmt1 | creatine kinase, mitochondrial 1, ubiquitous | 2,636196251 |
| 1434099_at | Ppargc1a | peroxisome proliferative activated receptor, gamma, coactivator 1 alpha | 2,585043923 |
| 1420672_at | Kcne1 | potassium voltage-gated channel, Isk-related subfamily, member 1 | 2,578370732 |
| 1418726_a_at | Tnnt2 | troponin T2, cardiac | 2,578315338 |
| 1434100_x_at | Ppargc1a | peroxisome proliferative activated receptor, gamma, coactivator 1 alpha | 2,535103647 |
| 1428667_at | Maoa | monoamine oxidase A | 2,518497657 |
| 1434025_at | --- | --- | 2,486545345 |
| 1438133_a_at | Cyr61 | cysteine rich protein 61 | 2,480072515 |
| 1437867_at | --- | --- | 2,479566105 |
| 1416023_at | Fabp3 | fatty acid binding protein 3, muscle and heart | 2,478853569 |
| 1422138_at | Plau | plasminogen activator, urokinase | 2,420362909 |
| 1451021_a_at | Klf5 | Kruppel-like factor 5 | 2,419124205 |
| 1418374_at | Fxyd3 | FXYD domain-containing ion transport regulator 3 | 2,412154027 |
| 1449464_at | Kcnq1 | potassium voltage-gated channel, subfamily Q, member 1 | 2,406525147 |
| 1451069_at | Pim3 | proviral integration site 3 | 2,369151078 |
| 1427195_at | Slc26a2 | solute carrier family 26 (sulfate transporter), member 2 | 2,368804268 |
| 1416761_at | Hsd11b2 | hydroxysteroid 11-beta dehydrogenase 2 | 2,311616406 |
| 1452406_x_at | Erdr1 | erythroid differentiation regulator 1 | 2,307292417 |
| 1417168_a_at | Usp2 | ubiquitin specific peptidase 2 | 2,284889961 |
| 1419884_at | --- | --- | 2,246739235 |
| 1460336_at | Ppargc1a | peroxisome proliferative activated receptor, gamma, coactivator 1 alpha | 2,240305451 |
| 1437100_x_at | Pim3 | proviral integration site 3 | 2,221834665 |
| 1418350_at | Hbegf | heparin-binding EGF-like growth factor | 2,194808473 |
| 1460250_at | Sostdc1 | sclerostin domain containing 1 | 2,183187173 |
| 1418709_at | Cox7a1 | cytochrome c oxidase, subunit VIIa 1 | 2,175268829 |
| 1425038_at | Slc22a19 | solute carrier family 22 (organic anion transporter), member 19 | 2,16540839 |
| 1415936_at | Bcar3 | breast cancer anti-estrogen resistance 3 | 2,164274521 |
| 1417158_at | Zxdc | ZXD family zinc finger C | 2,155916115 |
| 1449340_at | Sostdc1 | sclerostin domain containing 1 | 2,151372257 |
| 1460732_a_at | Ppl | periplakin | 2,1452282 |
| 1424265_at | Npl | N-acetylneuraminate pyruvate lyase | 2,144312462 |
| 1416039_x_at | Cyr61 | cysteine rich protein 61 | 2,140110451 |
| 1418349_at | Hbegf | heparin-binding EGF-like growth factor | 2,136714097 |
| 1452203_at | Obfc2a | oligonucleotide/oligosaccharide-binding fold containing 2A | 2,131174007 |
| 1416627_at | Spint1 | serine protease inhibitor, Kunitz type 1 | 2,123870586 |
| 1426960_a_at | Fa2h | fatty acid 2-hydroxylase | 2,104340261 |
| 1420018_s_at | Tspan8 | tetraspanin 8 | 2,103845536 |
| 1455106_a_at | Ckb | creatine kinase, brain | 2,087947695 |
| 1448660_at | Arhgdig | Rho GDP dissociation inhibitor (GDI) gamma | 2,081454979 |
| 1451780_at | Blnk | B-cell linker | 2,070977093 |
| 1416905_at | Guca2a | guanylate cyclase activator 2a (guanylin) | 2,066476687 |
| 1415837_at | Klk1 | kallikrein 1 | 2,065669405 |
| 1436926_at | Esrrb | estrogen related receptor, beta | 2,059363799 |
| 1443969_at | Irs2 | insulin receptor substrate 2 | 2,056069875 |
| 1447494_at | D7Bwg0826e | DNA segment, Chr 7, Brigham & Women's Genetics 0826 expressed | 2,051517331 |
| 1425900_at | Hkdc1 | hexokinase domain containing 1 | 2,042248892 |
| 1418697_at | Inmt | indolethylamine N-methyltransferase | 2,041030256 |
| 1417169_at | Usp2 | ubiquitin specific peptidase 2 | 2,035567555 |
| 1418025_at | Bhlhe40 | basic helix-loop-helix family, member e40 | 2,03490115 |
| 1419154_at | Tmprss2 | transmembrane protease, serine 2 | 2,033142959 |
| 1448107_x_at | Klk1 | kallikrein 1 | 2,013879344 |
| 1417362_at | Rhcg | Rhesus blood group-associated C glycoprotein | 1,998413356 |
| 1426600_at | Slc2a1 | solute carrier family 2 (facilitated glucose transporter), member 1 | 1,993388233 |
| 1423228_at | B4galt6 | UDP-Gal:betaGlcNAc beta 1,4-galactosyltransferase, polypeptide 6 | 1,981845155 |
| 1449369_at | Tmprss2 | transmembrane protease, serine 2 | 1,977691092 |
| 1421041_s_at | Gm3776 /// Gsta1 /// Gsta2 | predicted gene 3776 /// glutathione S-transferase, alpha 1 (Ya) /// glutathione S-transferase, alpha 2 (Yc2) | 1,9736842 |
| 1450188_s_at | Lipg | lipase, endothelial | 1,972914865 |
| 1434743_x_at | Rusc1 | RUN and SH3 domain containing 1 | 1,96703018 |
| 1415968_a_at | Kap | kidney androgen regulated protein | 1,946510511 |
| 1418681_at | Alg13 | asparagine-linked glycosylation 13 homolog (S. cerevisiae) | 1,94220212 |
| 1422170_at | Slc5a3 | solute carrier family 5 (inositol transporters), member 3 | 1,935436566 |
| 1421275_s_at | Socs4 | suppressor of cytokine signaling 4 | 1,92977124 |
| 1421943_at | Tgfa | transforming growth factor alpha | 1,9286888 |
| 1421262_at | Lipg | lipase, endothelial | 1,920941299 |
| 1439016_x_at | Sprr2a1 /// Sprr2a2 /// Sprr2a3 | small proline-rich protein 2A1 /// small proline-rich protein 2A2 /// small proline-rich protein 2A3 | 1,920474821 |
| 1426599_a_at | Slc2a1 | solute carrier family 2 (facilitated glucose transporter), member 1 | 1,919465454 |
| 1437062_s_at | Phyhipl | phytanoyl-CoA hydroxylase interacting protein-like | 1,910279735 |
| 1460521_a_at | Obfc2a | oligonucleotide/oligosaccharide-binding fold containing 2A | 1,906107673 |
| 1453851_a_at | Gadd45g | growth arrest and DNA-damage-inducible 45 gamma | 1,897961946 |
| 1437534_at | --- | --- | 1,894812147 |
| 1436014_a_at | Rusc1 | RUN and SH3 domain containing 1 | 1,886742528 |
| 1416916_at | Elf3 | E74-like factor 3 | 1,881188134 |
| 1423933_a_at | 1600029D21Rik | RIKEN cDNA 1600029D21 gene | 1,86882309 |
| 1418718_at | Cxcl16 | chemokine (C-X-C motif) ligand 16 | 1,86881804 |
| 1415969_s_at | Kap | kidney androgen regulated protein | 1,867968896 |
| 1416854_at | Slc34a2 | solute carrier family 34 (sodium phosphate), member 2 | 1,86077429 |
| 1420715_a_at | Pparg | peroxisome proliferator activated receptor gamma | 1,860486048 |
| 1451341_s_at | Tmem189 | transmembrane protein 189 | 1,856463424 |
| 1424649_a_at | Tspan8 | tetraspanin 8 | 1,856352329 |
| 1423968_at | Ugt3a2 | UDP glycosyltransferases 3 family, polypeptide A2 | 1,854437715 |
| 1456306_a_at | Umod | uromodulin | 1,853569141 |
| 1428742_at | Fbxo45 | F-box protein 45 | 1,846149874 |
| 1418923_at | Slc17a3 | solute carrier family 17 (sodium phosphate), member 3 | 1,845249783 |
| 1417616_at | St6galnac2 | ST6 (alpha-N-acetyl-neuraminyl-2,3-beta-galactosyl-1,3)-N-acetylgalactosaminide alpha-2,6-sialyltransferase 2 | 1,825672854 |
| 1419079_at | Scnn1g | sodium channel, nonvoltage-gated 1 gamma | 1,825208433 |
| 1418932_at | Nfil3 | nuclear factor, interleukin 3, regulated | 1,822181021 |
| 1452269_at | Spnb3 | spectrin beta 3 | 1,816869357 |
| 1427372_at | Cyp27b1 | cytochrome P450, family 27, subfamily b, polypeptide 1 | 1,814352102 |
| 1426808_at | Lgals3 | lectin, galactose binding, soluble 3 | 1,813958813 |
| 1421145_at | Slc26a2 | solute carrier family 26 (sulfate transporter), member 2 | 1,804784794 |
| 1426252_a_at | Umod | uromodulin | 1,80388172 |
| 1448397_at | Gjb6 | gap junction protein, beta 6 | 1,801824422 |
| 1420377_at | St8sia2 | ST8 alpha-N-acetyl-neuraminide alpha-2,8-sialyltransferase 2 | 1,800915998 |
| 1427749_at | Galnt3 | UDP-N-acetyl-alpha-D-galactosamine:polypeptide N-acetylgalactosaminyltransferase 3 | 1,797874419 |
| 1427243_at | Rell1 | RELT-like 1 | 1,792604305 |
| 1419473_a_at | Cck | cholecystokinin | 1,791243986 |
| 1426278_at | Ifi27l2a | interferon, alpha-inducible protein 27 like 2A | 1,787531818 |
| 1456190_a_at | Acsm2 | acyl-CoA synthetase medium-chain family member 2 | 1,783150054 |
| 1424208_at | Ptger4 | prostaglandin E receptor 4 (subtype EP4) | 1,780372429 |
| 1433579_at | Tmem30b | transmembrane protein 30B | 1,77731759 |
| 1453486_a_at | Scube2 | signal peptide, CUB domain, EGF-like 2 | 1,767389108 |
| 1451588_at | 1810022C23Rik | RIKEN cDNA 1810022C23 gene | 1,76241697 |
| 1425420_s_at | Lats2 | large tumor suppressor 2 | 1,761917857 |
| 1418488_s_at | Ripk4 | receptor-interacting serine-threonine kinase 4 | 1,760931481 |
| 1417072_at | Slc22a6 | solute carrier family 22 (organic anion transporter), member 6 | 1,7585834 |
| 1423952_a_at | Krt7 | keratin 7 | 1,756012278 |
| 1428046_a_at | Zfx | zinc finger protein X-linked | 1,755098 |
| 1425318_a_at | Tmem116 | transmembrane protein 116 | 1,754368081 |
| 1450264_a_at | Chka | choline kinase alpha | 1,752455023 |
| 1449773_s_at | Gadd45b | growth arrest and DNA-damage-inducible 45 beta | 1,749817412 |
| 1421377_at | Traf6 | TNF receptor-associated factor 6 | 1,747124786 |
| 1417392_a_at | Slc7a7 | solute carrier family 7 (cationic amino acid transporter, y+ system), member 7 | 1,742903609 |
| 1423590_at | Napsa | napsin A aspartic peptidase | 1,742836825 |
| 1437111_at | Zc3h12c | zinc finger CCCH type containing 12C | 1,737325187 |
| 1450936_a_at | Dnase1l2 | deoxyribonuclease 1-like 2 | 1,733809098 |
| 1419814_s_at | S100a1 | S100 calcium binding protein A1 | 1,731781091 |
| 1448741_at | Slc3a1 | solute carrier family 3, member 1 | 1,728457044 |
| 1418118_at | Slc22a1 | solute carrier family 22 (organic cation transporter), member 1 | 1,7273081 |
| 1417843_s_at | Eps8l2 | EPS8-like 2 | 1,725001575 |
| 1419729_at | Tex11 | testis expressed gene 11 | 1,722479341 |
| 1434773_a_at | Slc2a1 | solute carrier family 2 (facilitated glucose transporter), member 1 | 1,719268033 |
| 1424411_at | Tmem189 | transmembrane protein 189 | 1,71670093 |
| 1449851_at | Per1 | period homolog 1 (Drosophila) | 1,71472464 |
| 1417689_a_at | Pdzk1ip1 | PDZK1 interacting protein 1 | 1,705561433 |
| 1452359_at | Rell1 | RELT-like 1 | 1,702533665 |
| 1425001_at | Rnf146 | ring finger protein 146 | 1,700903945 |
| 1419117_at | Slc22a2 | solute carrier family 22 (organic cation transporter), member 2 | 1,698934513 |
| 1416432_at | Pfkfb3 | 6-phosphofructo-2-kinase/fructose-2,6-biphosphatase 3 | 1,698864526 |
| 1435951_at | Grip1 | glutamate receptor interacting protein 1 | 1,694336341 |
| 1417803_at | 1110032A04Rik | RIKEN cDNA 1110032A04 gene | 1,692432951 |
| 1425088_at | Scnn1a | sodium channel, nonvoltage-gated 1 alpha | 1,691893225 |
| 1427347_s_at | Tubb2a | tubulin, beta 2A | 1,688136162 |
| 1427878_at | 0610010O12Rik | RIKEN cDNA 0610010O12 gene | 1,68775612 |
| 1418910_at | Bmp7 | bone morphogenetic protein 7 | 1,684326317 |
| 1455372_at | Cpeb3 | cytoplasmic polyadenylation element binding protein 3 | 1,680285374 |
| 1418373_at | Pgam2 | phosphoglycerate mutase 2 | 1,678858084 |
| 1424549_at | Degs2 | degenerative spermatocyte homolog 2 (Drosophila), lipid desaturase | 1,674028844 |
| 1452270_s_at | Cubn | cubilin (intrinsic factor-cobalamin receptor) | 1,673895268 |
| 1425264_s_at | Mbp | myelin basic protein | 1,672599594 |
| 1438017_at | Rusc1 | RUN and SH3 domain containing 1 | 1,666250845 |
| 1452135_at | Gpx6 | glutathione peroxidase 6 | 1,664329005 |
| 1448506_at | Serpina6 | serine (or cysteine) peptidase inhibitor, clade A, member 6 | 1,660225382 |
| 1451258_at | Psca | prostate stem cell antigen | 1,660131161 |
| 1426990_at | Cubn | cubilin (intrinsic factor-cobalamin receptor) | 1,658777128 |
| 1419546_at | Atp6v1c1 | ATPase, H+ transporting, lysosomal V1 subunit C1 | 1,658752808 |
| 1448690_at | Kcnk1 | potassium channel, subfamily K, member 1 | 1,658469812 |
| 1426454_at | Arhgdib | Rho, GDP dissociation inhibitor (GDI) beta | 1,658437309 |
| 1424652_at | Fam176a | family with sequence similarity 176, member A | 1,65775442 |
| 1419545_a_at | Atp6v1c1 | ATPase, H+ transporting, lysosomal V1 subunit C1 | 1,656415183 |
| 1419314_at | Tinag | tubulointerstitial nephritis antigen | 1,654329244 |
| 1429239_a_at | Stard4 | StAR-related lipid transfer (START) domain containing 4 | 1,654132402 |
| 1428140_at | Oxct1 | 3-oxoacid CoA transferase 1 | 1,651102083 |
| 1455005_s_at | Slc5a2 | solute carrier family 5 (sodium/glucose cotransporter), member 2 | 1,650901913 |
| 1450135_at | Fzd3 | frizzled homolog 3 (Drosophila) | 1,650328884 |
| 1424960_at | Epn3 | epsin 3 | 1,64638154 |
| 1449409_at | Sult1c2 | sulfotransferase family, cytosolic, 1C, member 2 | 1,645976201 |
| 1427196_at | Wnk4 | WNK lysine deficient protein kinase 4 | 1,641842178 |
| 1435551_at | Fhod3 | formin homology 2 domain containing 3 | 1,640345185 |
| 1418449_at | Lad1 | ladinin | 1,639238442 |
| 1421073_a_at | Ptger4 | prostaglandin E receptor 4 (subtype EP4) | 1,636726233 |
| 1451635_at | C730048C13Rik /// D630002G06Rik | RIKEN cDNA C730048C13 gene /// RIKEN cDNA D630002G06 gene | 1,635202843 |
| 1421134_at | Areg | amphiregulin | 1,631011877 |
| 1451004_at | Acvr2a | activin receptor IIA | 1,629540983 |
| 1424638_at | Cdkn1a | cyclin-dependent kinase inhibitor 1A (P21) | 1,628361867 |
| 1423134_at | Rilpl2 | Rab interacting lysosomal protein-like 2 | 1,626966865 |
| 1451879_a_at | Slc6a18 | solute carrier family 6 (neurotransmitter transporter), member 18 | 1,624336571 |
| 1460248_at | Cpxm2 | carboxypeptidase X 2 (M14 family) | 1,611525394 |
| 1419447_s_at | Tbc1d1 | TBC1 domain family, member 1 | 1,611036175 |
| 1455281_at | Wdr33 | WD repeat domain 33 | 1,608963873 |
| 1448299_at | Slc1a1 | solute carrier family 1 (neuronal/epithelial high affinity glutamate transporter, system Xag), member 1 | 1,604984532 |
| 1427929_a_at | Pdxk | pyridoxal (pyridoxine, vitamin B6) kinase | 1,604644786 |
| 1427931_s_at | Pdxk | pyridoxal (pyridoxine, vitamin B6) kinase | 1,603986463 |
| 1451405_at | Pcca | propionyl-Coenzyme A carboxylase, alpha polypeptide | 1,603350725 |
| 1419195_at | Wfdc15b | WAP four-disulfide core domain 15B | 1,601907624 |
| 1417421_at | S100a1 | S100 calcium binding protein A1 | 1,599904195 |
| 1416893_at | Fam107b | family with sequence similarity 107, member B | 1,59961633 |
| 1450168_at | Ankrd12 | ankyrin repeat domain 12 | 1,599217835 |
| 1417047_at | Prom2 | prominin 2 | 1,599113071 |
| 1417920_at | Amn | amnionless | 1,598229077 |
| 1421040_a_at | Gsta2 | glutathione S-transferase, alpha 2 (Yc2) | 1,596535916 |
| 1424870_at | Osbpl10 | oxysterol binding protein-like 10 | 1,592095086 |
| 1418318_at | Rnf128 | ring finger protein 128 | 1,588310933 |
| 1428485_at | Car12 | carbonic anyhydrase 12 | 1,588302415 |
| 1418496_at | Foxa1 | forkhead box A1 | 1,586823956 |
| 1433775_at | C77080 | expressed sequence C77080 | 1,58603719 |
| 1427235_at | Kdm6a | 4lysine (K)-specific demethylase 6A | 1,58385317 |
| 1423279_at | Slc34a1 | solute carrier family 34 (sodium phosphate), member 1 | 1,580081371 |
| 1425855_a_at | Crk | v-crk sarcoma virus CT10 oncogene homolog (avian) | 1,579433149 |
| 1425536_at | Stx3 | syntaxin 3 | 1,576796896 |
| 1418468_at | Anxa11 | annexin A11 | 1,576283207 |
| 1449059_a_at | Oxct1 | 3-oxoacid CoA transferase 1 | 1,574787759 |
| 1425282_at | Rnf144b | ring finger protein 144B | 1,570457769 |
| 1431805_a_at | Rhpn2 | rhophilin, Rho GTPase binding protein 2 | 1,569757957 |
| 1450251_a_at | Lnx1 | ligand of numb-protein X 1 | 1,568958363 |
| 1417293_at | Hs6st1 | heparan sulfate 6-O-sulfotransferase 1 | 1,567520407 |
| 1435758_at | B4galt6 | UDP-Gal:betaGlcNAc beta 1,4-galactosyltransferase, polypeptide 6 | 1,566798621 |
| 1418966_a_at | Dcbld1 | discoidin, CUB and LCCL domain containing 1 | 1,56628731 |
| 1427672_a_at | Kdm6a | 4lysine (K)-specific demethylase 6A | 1,565386988 |
| 1426980_s_at | E130012A19Rik | RIKEN cDNA E130012A19 gene | 1,564826799 |
| 1421947_at | Gng12 | guanine nucleotide binding protein (G protein), gamma 12 | 1,564567607 |
| 1449336_a_at | Slk | STE20-like kinase (yeast) | 1,563477466 |
| 1455825_s_at | Lnx1 | ligand of numb-protein X 1 | 1,561395358 |
| 1454268_a_at | Cyba | cytochrome b-245, alpha polypeptide | 1,561346983 |
| 1450719_at | Mep1a | meprin 1 alpha | 1,560718264 |
| 1418829_a_at | Eno2 | enolase 2, gamma neuronal | 1,560143099 |
| 1419766_at | Sik1 | salt inducible kinase 1 | 1,559291429 |
| 1451765_a_at | Entpd5 | ectonucleoside triphosphate diphosphohydrolase 5 | 1,556423224 |
| 1420760_s_at | Ndrg1 | N-myc downstream regulated gene 1 | 1,553859929 |
| 1423174_a_at | Pard6b | par-6 (partitioning defective 6) homolog beta (C. elegans) | 1,552204135 |
| 1419490_at | Fam19a5 | family with sequence similarity 19, member A5 | 1,551415718 |
| 1455089_at | Gng12 | guanine nucleotide binding protein (G protein), gamma 12 | 1,550433589 |
| 1421595_at | Fam184b | family with sequence similarity 184, member B | 1,55013872 |
| 1426014_a_at | Cdhr5 | cadherin-related family member 5 | 1,547905147 |
| 1428736_at | Gramd3 | GRAM domain containing 3 | 1,546941031 |
| 1425792_a_at | Rorc | RAR-related orphan receptor gamma | 1,544902166 |
| 1419379_x_at | Fxyd2 | FXYD domain-containing ion transport regulator 2 | 1,544756699 |
| 1451515_s_at | Glyat | glycine-N-acyltransferase | 1,544684721 |
| 1423323_at | Tacstd2 | tumor-associated calcium signal transducer 2 | 1,539939212 |
| 1421821_at | Ldlr | low density lipoprotein receptor | 1,538410297 |
| 1449458_at | Foxi1 | forkhead box I1 | 1,537342347 |
| 1448783_at | Slc7a9 | solute carrier family 7 (cationic amino acid transporter, y+ system), member 9 | 1,53692981 |
| 1416199_at | Kifc3 | kinesin family member C3 | 1,536619403 |
| 1419446_at | Tbc1d1 | TBC1 domain family, member 1 | 1,536563097 |
| 1419134_at | Rhbg | Rhesus blood group-associated B glycoprotein | 1,53509107 |
| 1418215_at | Mep1b | meprin 1 beta | 1,534849751 |
| 1455477_s_at | Pdzk1ip1 | PDZK1 interacting protein 1 | 1,532359167 |
| 1425400_a_at | Cited4 | Cbp/p300-interacting transactivator, with Glu/Asp-rich carboxy-terminal domain, 4 | 1,529876977 |
| 1423434_at | Tead1 | TEA domain family member 1 | 1,529104095 |
| 1435077_at | Asxl1 | additional sex combs like 1 (Drosophila) | 1,524888562 |
| 1460329_at | B4galt6 | UDP-Gal:betaGlcNAc beta 1,4-galactosyltransferase, polypeptide 6 | 1,52241498 |
| 1427308_at | Dab1 | disabled homolog 1 (Drosophila) | 1,519757987 |
| 1421274_at | Socs4 | suppressor of cytokine signaling 4 | 1,519141195 |
| 1423854_a_at | Rasl11b | RAS-like, family 11, member B | 1,515586092 |
| 1418059_at | Eltd1 | EGF, latrophilin seven transmembrane domain containing 1 | 1,514976878 |
| 1451090_a_at | Eif2s3x | eukaryotic translation initiation factor 2, subunit 3, structural gene X-linked | 1,511585162 |
| 1418723_at | Lpar3 | lysophosphatidic acid receptor 3 | 1,510788602 |
| 1439375_x_at | Aldoa | aldolase A, fructose-bisphosphate | 1,509770086 |
| 1455896_a_at | Kcnk1 | potassium channel, subfamily K, member 1 | 1,509632604 |
| 1452321_at | Brwd1 | bromodomain and WD repeat domain containing 1 | 1,509362851 |
| 1426146_a_at | Chpt1 | choline phosphotransferase 1 | 1,508089686 |
| 1450070_s_at | Pak1 | p21 protein (Cdc42/Rac)-activated kinase 1 | 1,507716537 |
| 1423555_a_at | Ifi44 | interferon-induced protein 44 | 1,506206561 |
| 1421422_at | 5033411D12Rik | RIKEN cDNA 5033411D12 gene | 1,504824957 |
| 1453470_a_at | Gna13 | guanine nucleotide binding protein, alpha 13 | 1,504591673 |
| 1431701_a_at | Pdzk1 | PDZ domain containing 1 | 1,502038885 |
| 1420425_at | Prdm1 | PR domain containing 1, with ZNF domain | 1,501698565 |
| 1427838_at | Tubb2a | tubulin, beta 2A | 1,500660327 |
| 1420573_at | Hoxd1 | homeobox D1 | 1,50051845 |
| 1431711_a_at | 9030409G11Rik | RIKEN cDNA 9030409G11 gene | 1,500289134 |
| 1450704_at | Ihh | Indian hedgehog | 1,4930797 |
| 1426461_at | Ugp2 | UDP-glucose pyrophosphorylase 2 | 1,491547596 |
| 1423175_s_at | Pard6b | par-6 (partitioning defective 6) homolog beta (C. elegans) | 1,490399498 |
| 1448118_a_at | Ctsd | cathepsin D | 1,489617098 |
| 1417812_a_at | Lamb3 | laminin, beta 3 | 1,486301081 |
| 1426020_at | Tmpo | thymopoietin | 1,483486856 |
| 1449106_at | Gpx3 | glutathione peroxidase 3 | 1,482844416 |
| 1456174_x_at | Ndrg1 | N-myc downstream regulated gene 1 | 1,481033756 |
| 1450622_at | Bcar1 | breast cancer anti-estrogen resistance 1 | 1,470751034 |
| 1460341_at | Plekhb2 | pleckstrin homology domain containing, family B (evectins) member 2 | 1,470495141 |
| 1436890_at | Uap1l1 | UDP-N-acteylglucosamine pyrophosphorylase 1-like 1 | 1,469342232 |
| 1417810_a_at | Kcnb1 /// Pacsin2 | potassium voltage gated channel, Shab-related subfamily, member 1 /// protein kinase C and casein kinase substrate in neurons 2 | 1,469101821 |
| 1416617_at | Acss1 | acyl-CoA synthetase short-chain family member 1 | 1,467642657 |
| 1448883_at | Lgmn | legumain | 1,464173734 |
| 1451379_at | Rab22a | RAB22A, member RAS oncogene family | 1,46065946 |
| 1423519_at | Fam108c | family with sequence similarity 108, member C | 1,458890818 |
| 1417818_at | Wwtr1 | WW domain containing transcription regulator 1 | 1,455721655 |
| 1452050_at | Camk1d | calcium/calmodulin-dependent protein kinase ID | 1,45528198 |
| 1449205_at | Ovol2 | ovo-like 2 (Drosophila) | 1,454786605 |
| 1416046_a_at | Fuca2 | fucosidase, alpha-L- 2, plasma | 1,450840902 |
| 1419544_at | Atp6v1c1 | ATPase, H+ transporting, lysosomal V1 subunit C1 | 1,450559466 |
| 1451002_at | Aco2 | aconitase 2, mitochondrial | 1,449053475 |
| 1422823_at | Eps8 | epidermal growth factor receptor pathway substrate 8 | 1,437318355 |
| 1419695_at | St8sia1 | ST8 alpha-N-acetyl-neuraminide alpha-2,8-sialyltransferase 1 | 1,43115602 |
| 1437991_x_at | Rusc1 | RUN and SH3 domain containing 1 | 1,423423598 |
| 1455126_x_at | 2310028O11Rik | RIKEN cDNA 2310028O11 gene | 1,422211959 |
| 1425582_a_at | Emcn | endomucin | 1,415604368 |
| 1448787_at | LOC100233175 /// Moap1 | hypothetical protein LOC100233175 /// modulator of apoptosis 1 | 1,415319863 |
| 1421455_at | Sntb1 | syntrophin, basic 1 | 1,414066901 |
| 1449317_at | Cflar | CASP8 and FADD-like apoptosis regulator | 1,413887644 |
| 1434799_x_at | Aldoa | aldolase A, fructose-bisphosphate | 1,407777408 |
| 1452233_at | Abcc1 | ATP-binding cassette, sub-family C (CFTR/MRP), member 1 | 1,399740961 |
| 1427629_at | Ptprj | protein tyrosine phosphatase, receptor type, J | 1,398388119 |
| 1424351_at | Wfdc2 | WAP four-disulfide core domain 2 | 1,395499128 |
| 1422483_a_at | Cycs | cytochrome c, somatic | 1,393824522 |
| 1423561_at | Nell2 | NEL-like 2 (chicken) | 1,393624523 |
| 1419378_a_at | Fxyd2 | FXYD domain-containing ion transport regulator 2 | 1,393094431 |
| 1418115_s_at | Tor1aip2 | torsin A interacting protein 2 | 1,391557276 |
| 1425640_at | Aff1 | AF4/FMR2 family, member 1 | 1,390763037 |
| 1429745_at | DXBay18 /// Gm14685 /// Gm5640 /// Gm5936 | DNA segment, Chr X, Baylor 18 /// predicted gene 14685 /// predicted gene 5640 /// predicted gene 5936 | 1,390306758 |
| 1436934_s_at | Aco2 | aconitase 2, mitochondrial | 1,383573498 |
| 1418104_at | Nrip3 | nuclear receptor interacting protein 3 | 1,38179972 |
| 1424704_at | Runx2 | runt related transcription factor 2 | 1,381095444 |
| 1448698_at | Ccnd1 | cyclin D1 | 1,377411994 |
| 1426800_at | Rab8b | RAB8B, member RAS oncogene family | 1,377332787 |
| 1416921_x_at | Aldoa | aldolase A, fructose-bisphosphate | 1,374979506 |
| 1422525_at | Atp5k | ATP synthase, H+ transporting, mitochondrial F1F0 complex, subunit e | 1,373393455 |
| 1437583_x_at | Acss1 | acyl-CoA synthetase short-chain family member 1 | 1,369381102 |
| 1459983_at | Impa2 | inositol (myo)-1(or 4)-monophosphatase 2 | 1,368823874 |
| 1448237_x_at | Ldhb | lactate dehydrogenase B | 1,368313208 |
| 1449254_at | Spp1 | secreted phosphoprotein 1 | 1,361673498 |
| 1456601_x_at | Fxyd2 | FXYD domain-containing ion transport regulator 2 | 1,345055587 |
| 1431719_a_at | Srgap1 | SLIT-ROBO Rho GTPase activating protein 1 | 1,344627984 |
| 1454890_at | Amot | angiomotin | 1,341768898 |
| 1450640_x_at | Atp5k | ATP synthase, H+ transporting, mitochondrial F1F0 complex, subunit e | 1,341341241 |
| 1449650_at | --- | --- | 1,336982185 |
| 1422578_at | Cs | citrate synthase | 1,335630501 |
| 1431619_a_at | Dtnbp1 | dystrobrevin binding protein 1 | 1,33057279 |
| 1422226_at | --- | --- | 1,328425999 |
| 1426776_at | Wasl | Wiskott-Aldrich syndrome-like (human) | 1,327665844 |
| 1416285_at | Ndufc1 | NADH dehydrogenase (ubiquinone) 1, subcomplex unknown, 1 | 1,322111475 |
| 1455235_x_at | Ldhb | lactate dehydrogenase B | 1,320185537 |
| 1416268_at | Ets2 | E26 avian leukemia oncogene 2, 3' domain | 1,320101197 |
| 1425198_at | Ptpn2 | protein tyrosine phosphatase, non-receptor type 2 | 1,316814632 |
| 1450626_at | Manba | mannosidase, beta A, lysosomal | 1,308092547 |
| 1448349_at | Vapa | vesicle-associated membrane protein, associated protein A | 1,308050326 |
| 1431981_at | Hif1a | hypoxia inducible factor 1, alpha subunit | 1,307964896 |
| 1424052_at | Thap4 | THAP domain containing 4 | 1,30614003 |
| 1417185_at | Ly6a | lymphocyte antigen 6 complex, locus A | 1,2993303 |
| 1416041_at | Sgk1 | serum/glucocorticoid regulated kinase 1 | 1,299210793 |
| 1427404_x_at | Gm5506 | predicted gene 5506 | 1,292523572 |
| 1416565_at | Cox6b1 | cytochrome c oxidase, subunit VIb polypeptide 1 | 1,292304853 |
| 1448022_at | --- | --- | 1,287829728 |
| 1415970_at | Cox6c | cytochrome c oxidase, subunit VIc | 1,287770568 |
| 1423890_x_at | Atp1b1 | ATPase, Na+/K+ transporting, beta 1 polypeptide | 1,28525554 |
| 1432479_at | Spn-ps | sialophorin, pseudogene | 1,278713916 |
| 1456002_at | Xpa | xeroderma pigmentosum, complementation group A | 1,278419031 |
| 1456588_x_at | Cox5b | cytochrome c oxidase, subunit Vb | 1,206573644 |
| 1435613_x_at | Cox5b | cytochrome c oxidase, subunit Vb | 1,205976331 |
| 1419600_at | Defb4 | defensin beta 4 | 1,196120767 |
| 1417417_a_at | Cox6a1 | cytochrome c oxidase, subunit VI a, polypeptide 1 | 1,182601501 |
| 1421165_at | Mycbp | c-myc binding protein | 1,134725874 |
