# Supplementary material for: Modelling TFE renal cell carcinoma in mice reveals a critical role of WNT signaling
Source: eLife. 2016 Sep 26;5:e17047. doi: 10.7554/eLife.17047 (PMC5036965; doi:10.7554/eLife.17047)
Supplement: Figure 3—source data 2. — The genes are ranked by decreasing signed ratio (KSP_P14/CTL). DOI: http://dx.doi.org/10.7554/eLife.17047.009 [file elife-17047-fig3-data2.docx]

| **Probe Set ID** | **Gene Symbol** | **Gene Title** | **signed_ratio (KSP_P14/CTL)** |
| --- | --- | --- | --- |
| 1448303_at | Gpnmb | glycoprotein (transmembrane) nmb | 4,926015853 |
| 1418866_at | Cyp24a1 | cytochrome P450, family 24, subfamily a, polypeptide 1 | 4,817745787 |
| 1448660_at | Arhgdig | Rho GDP dissociation inhibitor (GDI) gamma | 4,712974462 |
| 1416905_at | Guca2a | guanylate cyclase activator 2a (guanylin) | 3,821491961 |
| 1449475_at | Atp12a | ATPase, H+/K+ transporting, nongastric, alpha polypeptide | 3,410462324 |
| 1449896_at | Mlph | melanophilin | 3,345988188 |
| 1416523_at | Rnase1 | ribonuclease, RNase A family, 1 (pancreatic) | 3,27990729 |
| 1421286_a_at | Atp4a | ATPase, H+/K+ exchanging, gastric, alpha polypeptide | 2,706859806 |
| 1434743_x_at | Rusc1 | RUN and SH3 domain containing 1 | 2,693050655 |
| 1422552_at | Rprm | reprimo, TP53 dependent G2 arrest mediator candidate | 2,636325805 |
| 1442028_at | B4galnt2 | beta-1,4-N-acetyl-galactosaminyl transferase 2 | 2,594523609 |
| 1425245_a_at | Rgs11 | regulator of G-protein signaling 11 | 2,519093928 |
| 1449833_at | Sprr2f | small proline-rich protein 2F | 2,485821887 |
| 1449166_at | S100a14 | S100 calcium binding protein A14 | 2,439748939 |
| 1418412_at | Tpd52l1 | tumor protein D52-like 1 | 2,381774817 |
| 1448954_at | Nrip3 | nuclear receptor interacting protein 3 | 2,338517527 |
| 1436014_a_at | Rusc1 | RUN and SH3 domain containing 1 | 2,27819033 |
| 1425400_a_at | Cited4 | Cbp/p300-interacting transactivator, with Glu/Asp-rich carboxy-terminal domain, 4 | 2,275643713 |
| 1452269_at | Spnb3 | spectrin beta 3 | 2,258739516 |
| 1416464_at | Slc4a1 | solute carrier family 4 (anion exchanger), member 1 | 2,183587743 |
| 1421113_at | Pga5 | pepsinogen 5, group I | 2,144909532 |
| 1435939_s_at | Hepacam2 | HEPACAM family member 2 | 2,098301038 |
| 1436912_at | Cacnb4 | calcium channel, voltage-dependent, beta 4 subunit | 2,069952296 |
| 1449464_at | Kcnq1 | potassium voltage-gated channel, subfamily Q, member 1 | 2,062841283 |
| 1427537_at | Eppk1 | epiplakin 1 | 2,049143198 |
| 1423933_a_at | 1600029D21Rik | RIKEN cDNA 1600029D21 gene | 2,033161001 |
| 1425153_at | Myh2 | myosin, heavy polypeptide 2, skeletal muscle, adult | 2,023045344 |
| 1449555_a_at | Fetub | fetuin beta | 1,994578837 |
| 1434502_x_at | Slc4a1 | solute carrier family 4 (anion exchanger), member 1 | 1,990340937 |
| 1460230_at | Syn2 | synapsin II | 1,978507596 |
| 1417089_a_at | Ckmt1 | creatine kinase, mitochondrial 1, ubiquitous | 1,961146631 |
| 1450344_a_at | Ptger3 | prostaglandin E receptor 3 (subtype EP3) | 1,957736919 |
| 1418475_at | Scnn1b | sodium channel, nonvoltage-gated 1 beta | 1,955879846 |
| 1419666_x_at | Nupr1 | nuclear protein 1 | 1,95386887 |
| 1417362_at | Rhcg | Rhesus blood group-associated C glycoprotein | 1,951412851 |
| 1418282_x_at | Serpina1b | serine (or cysteine) preptidase inhibitor, clade A, member 1B | 1,945165644 |
| 1433919_at | Asb4 | ankyrin repeat and SOCS box-containing 4 | 1,93806545 |
| 1419665_a_at | Nupr1 | nuclear protein 1 | 1,915292357 |
| 1419147_at | Rec8 | REC8 homolog (yeast) | 1,909654444 |
| 1415856_at | Emb | embigin | 1,893315033 |
| 1422324_a_at | Pthlh | parathyroid hormone-like peptide | 1,877006191 |
| 1436021_at | Mfsd4 | major facilitator superfamily domain containing 4 | 1,873335826 |
| 1443969_at | Irs2 | insulin receptor substrate 2 | 1,870668787 |
| 1424549_at | Degs2 | degenerative spermatocyte homolog 2 (Drosophila), lipid desaturase | 1,848980453 |
| 1448502_at | Slc16a7 | solute carrier family 16 (monocarboxylic acid transporters), member 7 | 1,839866297 |
| 1421145_at | Slc26a2 | solute carrier family 26 (sulfate transporter), member 2 | 1,834333842 |
| 1427344_s_at | Rasd2 | RASD family, member 2 | 1,830811429 |
| 1420018_s_at | Tspan8 | tetraspanin 8 | 1,825959984 |
| 1451513_x_at | Serpina1a /// Serpina1b | serine (or cysteine) peptidase inhibitor, clade A, member 1A /// serine (or cysteine) preptidase inhibitor, clade A, member 1B | 1,808595399 |
| 1448327_at | Actn2 | actinin alpha 2 | 1,80859156 |
| 1454736_at | Ankrd57 | ankyrin repeat domain 57 | 1,799894808 |
| 1455106_a_at | Ckb | creatine kinase, brain | 1,795327127 |
| 1425109_at | Slc44a3 | solute carrier family 44, member 3 | 1,794600259 |
| 1419083_at | Tnfsf11 | tumor necrosis factor (ligand) superfamily, member 11 | 1,786214735 |
| 1420399_at | Gfi1b | growth factor independent 1B | 1,781262831 |
| 1418076_at | St14 | suppression of tumorigenicity 14 (colon carcinoma) | 1,779929616 |
| 1415857_at | Emb | embigin | 1,779034891 |
| 1449458_at | Foxi1 | forkhead box I1 | 1,764773068 |
| 1416761_at | Hsd11b2 | hydroxysteroid 11-beta dehydrogenase 2 | 1,760868762 |
| 1424933_at | Myo5c | myosin VC | 1,757114875 |
| 1416649_at | Ambp | alpha 1 microglobulin/bikunin | 1,750115452 |
| 1421996_at | Tcfap2a | transcription factor AP-2, alpha | 1,733153092 |
| 1443695_at | Habp2 | hyaluronic acid binding protein 2 | 1,732568084 |
| 1449273_at | Cyfip2 | cytoplasmic FMR1 interacting protein 2 | 1,709209164 |
| 1426566_s_at | Il17re | interleukin 17 receptor E | 1,705090057 |
| 1419332_at | Egfl6 | EGF-like-domain, multiple 6 | 1,697572247 |
| 1425804_at | Hmx2 | H6 homeobox 2 | 1,696772454 |
| 1434199_at | Tmem151b | transmembrane protein 151B | 1,674397907 |
| 1438017_at | Rusc1 | RUN and SH3 domain containing 1 | 1,670742455 |
| 1437991_x_at | Rusc1 | RUN and SH3 domain containing 1 | 1,664927768 |
| 1416361_a_at | Dync1i1 | dynein cytoplasmic 1 intermediate chain 1 | 1,664819798 |
| 1427838_at | Tubb2a | tubulin, beta 2A | 1,656533377 |
| 1448301_s_at | Serpinb1a | serine (or cysteine) peptidase inhibitor, clade B, member 1a | 1,656456742 |
| 1448507_at | Efhd1 | EF hand domain containing 1 | 1,649144622 |
| 1420762_a_at | Ybx2 | Y box protein 2 | 1,645307186 |
| 1425357_a_at | Grem1 | gremlin 1 | 1,640123107 |
| 1449028_at | Rhou | ras homolog gene family, member U | 1,639718601 |
| 1427347_s_at | Tubb2a | tubulin, beta 2A | 1,63858201 |
| 1420017_at | Tspan8 | tetraspanin 8 | 1,629907053 |
| 1428667_at | Maoa | monoamine oxidase A | 1,629156252 |
| 1424960_at | Epn3 | epsin 3 | 1,626959106 |
| 1425264_s_at | Mbp | myelin basic protein | 1,626772951 |
| 1418603_at | Avpr1a | arginine vasopressin receptor 1A | 1,626642382 |
| 1435697_a_at | Cytip | cytohesin 1 interacting protein | 1,621666971 |
| 1418373_at | Pgam2 | phosphoglycerate mutase 2 | 1,614831028 |
| 1420672_at | Kcne1 | potassium voltage-gated channel, Isk-related subfamily, member 1 | 1,611095845 |
| 1424474_a_at | Camkk2 | calcium/calmodulin-dependent protein kinase kinase 2, beta | 1,610472443 |
| 1427195_at | Slc26a2 | solute carrier family 26 (sulfate transporter), member 2 | 1,606600198 |
| 1426926_at | Plcg2 | phospholipase C, gamma 2 | 1,601227563 |
| 1416646_at | Afp | alpha fetoprotein | 1,600726946 |
| 1449321_x_at | Serpina1a /// Serpina1b /// Serpina1c /// Serpina1d /// Serpina1e | serine (or cysteine) peptidase inhibitor, clade A, member 1A /// serine (or cysteine) preptidase inhibitor, clade A, member 1B /// serine (or cysteine) peptidase inhibitor, clade A, member 1C /// serine (or cysteine) peptidase inhibitor, clade A, member 1D /// serine (or cysteine) peptidase inhibitor, clade A, member 1E | 1,599412667 |
| 1451693_a_at | Fgf12 | fibroblast growth factor 12 | 1,597033193 |
| 1416645_a_at | Afp | alpha fetoprotein | 1,595799194 |
| 1418468_at | Anxa11 | annexin A11 | 1,587821493 |
| 1450781_at | Hmga2 | high mobility group AT-hook 2 | 1,581850767 |
| 1426302_at | Tmprss4 | transmembrane protease, serine 4 | 1,578781372 |
| 1419079_at | Scnn1g | sodium channel, nonvoltage-gated 1 gamma | 1,577878809 |
| 1426517_at | Gnaz | guanine nucleotide binding protein, alpha z subunit | 1,575435765 |
| 1423228_at | B4galt6 | UDP-Gal:betaGlcNAc beta 1,4-galactosyltransferase, polypeptide 6 | 1,574915285 |
| 1424653_at | Tspan15 | tetraspanin 15 | 1,571157733 |
| 1418655_at | B4galnt1 | beta-1,4-N-acetyl-galactosaminyl transferase 1 | 1,570192896 |
| 1419755_at | Mfi2 | antigen p97 (melanoma associated) identified by monoclonal antibodies 133.2 and 96.5 | 1,565849006 |
| 1422643_at | Moxd1 | monooxygenase, DBH-like 1 | 1,564608641 |
| 1423422_at | Asb4 | ankyrin repeat and SOCS box-containing 4 | 1,56437722 |
| 1427912_at | Cbr3 | carbonyl reductase 3 | 1,560381448 |
| 1449027_at | Rhou | ras homolog gene family, member U | 1,557669896 |
| 1451780_at | Blnk | B-cell linker | 1,556992107 |
| 1420630_at | 8430419L09Rik | RIKEN cDNA 8430419L09 gene | 1,551755731 |
| 1437358_at | Wdfy1 | WD repeat and FYVE domain containing 1 | 1,549791672 |
| 1448568_a_at | Slc20a1 | solute carrier family 20, member 1 | 1,548258896 |
| 1422601_at | Serpinb9 | serine (or cysteine) peptidase inhibitor, clade B, member 9 | 1,547137579 |
| 1452089_at | Cacnb4 | calcium channel, voltage-dependent, beta 4 subunit | 1,545725304 |
| 1425586_a_at | Mlph | melanophilin | 1,545561193 |
| 1420019_at | Tspan8 | Tetraspanin 8 | 1,543696746 |
| 1419615_at | Trpv6 | transient receptor potential cation channel, subfamily V, member 6 | 1,543186282 |
| 1416916_at | Elf3 | E74-like factor 3 | 1,538657999 |
| 1431434_at | Stk39 | serine/threonine kinase 39, STE20/SPS1 homolog (yeast) | 1,536079427 |
| 1415936_at | Bcar3 | breast cancer anti-estrogen resistance 3 | 1,535030124 |
| 1435758_at | B4galt6 | UDP-Gal:betaGlcNAc beta 1,4-galactosyltransferase, polypeptide 6 | 1,534422956 |
| 1418283_at | Cldn4 | claudin 4 | 1,532506906 |
| 1449199_at | Muc1 | mucin 1, transmembrane | 1,529516562 |
| 1425088_at | Scnn1a | sodium channel, nonvoltage-gated 1 alpha | 1,5275839 |
| 1436879_x_at | Afp | alpha fetoprotein | 1,525283111 |
| 1417889_at | Apobec2 | apolipoprotein B mRNA editing enzyme, catalytic polypeptide 2 | 1,525056017 |
| 1426867_at | Dmrt2 | doublesex and mab-3 related transcription factor 2 | 1,524986217 |
| 1416318_at | Serpinb1a | serine (or cysteine) peptidase inhibitor, clade B, member 1a | 1,519684123 |
| 1426875_s_at | Srxn1 | sulfiredoxin 1 homolog (S. cerevisiae) | 1,517167419 |
| 1419373_at | Atp6v1b1 | ATPase, H+ transporting, lysosomal V1 subunit B1 | 1,515256237 |
| 1422153_a_at | Asb11 | ankyrin repeat and SOCS box-containing 11 | 1,513298375 |
| 1439018_at | Fhdc1 | FH2 domain containing 1 | 1,51180853 |
| 1420564_at | Insrr | insulin receptor-related receptor | 1,511520644 |
| 1449890_at | Ugt2b37 | UDP glucuronosyltransferase 2 family, polypeptide B37 | 1,506046691 |
| 1424749_at | Wdfy1 | WD repeat and FYVE domain containing 1 | 1,492541574 |
| 1425704_at | Dhrs11 | dehydrogenase/reductase (SDR family) member 11 | 1,491447091 |
| 1419959_s_at | Cphx | cytoplasmic polyadenylated homeobox | 1,491086238 |
| 1452367_at | Coro2a | coronin, actin binding protein 2A | 1,490011917 |
| 1427772_at | Defb15 | defensin beta 15 | 1,486370742 |
| 1449880_s_at | Bglap /// Bglap-rs1 /// Bglap2 | bone gamma carboxyglutamate protein /// bone gamma-carboxyglutamate protein, related sequence 1 /// bone gamma-carboxyglutamate protein 2 | 1,485775095 |
| 1417169_at | Usp2 | ubiquitin specific peptidase 2 | 1,482521968 |
| 1430306_a_at | Atp6v1c2 | ATPase, H+ transporting, lysosomal V1 subunit C2 | 1,4814873 |
| 1424522_at | Heatr1 | HEAT repeat containing 1 | 1,477327098 |
| 1421963_a_at | Cdc25b | cell division cycle 25 homolog B (S. pombe) | 1,477046749 |
| 1418744_s_at | Tesc | tescalcin | 1,476996002 |
| 1418449_at | Lad1 | ladinin | 1,472082976 |
| 1448118_a_at | Ctsd | cathepsin D | 1,470577239 |
| 1419067_a_at | Rabgef1 | RAB guanine nucleotide exchange factor (GEF) 1 | 1,469193229 |
| 1450340_a_at | Clcnkb | chloride channel Kb | 1,46725451 |
| 1425312_s_at | Cpsf4l | cleavage and polyadenylation specific factor 4-like | 1,463332106 |
| 1427052_at | Acacb | acetyl-Coenzyme A carboxylase beta | 1,462552457 |
| 1417561_at | Apoc1 | apolipoprotein C-I | 1,45731224 |
| 1418957_at | Stac | src homology three (SH3) and cysteine rich domain | 1,456891602 |
| 1425274_at | Asph | aspartate-beta-hydroxylase | 1,456761796 |
| 1442531_at | D12Ertd123e | DNA segment, Chr 12, ERATO Doi 123, expressed | 1,456470199 |
| 1460329_at | B4galt6 | UDP-Gal:betaGlcNAc beta 1,4-galactosyltransferase, polypeptide 6 | 1,453525763 |
| 1423952_a_at | Krt7 | keratin 7 | 1,452944427 |
| 1436872_at | Tacc3 | transforming, acidic coiled-coil containing protein 3 | 1,451486477 |
| 1451320_at | Arhgap8 | Rho GTPase activating protein 8 | 1,450682048 |
| 1423627_at | Nqo1 | NAD(P)H dehydrogenase, quinone 1 | 1,450159871 |
| 1417896_at | Tjp3 | tight junction protein 3 | 1,449478456 |
| 1424930_s_at | Fam83f | family with sequence similarity 83, member F | 1,44777828 |
| 1417168_a_at | Usp2 | ubiquitin specific peptidase 2 | 1,447771497 |
| 1428485_at | Car12 | carbonic anyhydrase 12 | 1,445784762 |
| 1455825_s_at | Lnx1 | ligand of numb-protein X 1 | 1,445243689 |
| 1417551_at | Cln3 | ceroid lipofuscinosis, neuronal 3, juvenile (Batten, Spielmeyer-Vogt disease) | 1,445087427 |
| 1431829_a_at | Rgl3 | ral guanine nucleotide dissociation stimulator-like 3 | 1,443043262 |
| 1419489_at | Fam19a5 | family with sequence similarity 19, member A5 | 1,441793015 |
| 1427196_at | Wnk4 | WNK lysine deficient protein kinase 4 | 1,436127825 |
| 1430798_x_at | Mrpl15 | mitochondrial ribosomal protein L15 | 1,434707988 |
| 1422754_at | Tmod1 | tropomodulin 1 | 1,433180633 |
| 1424838_at | A330049M08Rik | RIKEN cDNA A330049M08 gene | 1,43304266 |
| 1434100_x_at | Ppargc1a | peroxisome proliferative activated receptor, gamma, coactivator 1 alpha | 1,432498119 |
| 1449107_at | Nudt4 | nudix (nucleoside diphosphate linked moiety X)-type motif 4 | 1,430294937 |
| 1426656_at | Fam63a | family with sequence similarity 63, member A | 1,429702398 |
| 1416820_at | Cdc37 | cell division cycle 37 homolog (S. cerevisiae) | 1,428839363 |
| 1417587_at | Timeless | timeless homolog (Drosophila) | 1,426152636 |
| 1424759_at | Arrdc4 | arrestin domain containing 4 | 1,42465496 |
| 1434927_at | Hspb7 | heat shock protein family, member 7 (cardiovascular) | 1,423882834 |
| 1434099_at | Ppargc1a | peroxisome proliferative activated receptor, gamma, coactivator 1 alpha | 1,423033116 |
| 1415810_at | Uhrf1 | ubiquitin-like, containing PHD and RING finger domains, 1 | 1,421712943 |
| 1429347_at | Bcl2l14 | BCL2-like 14 (apoptosis facilitator) | 1,421548897 |
| 1426048_s_at | Tcfap2a | transcription factor AP-2, alpha | 1,421318683 |
| 1454837_at | Cln6 | ceroid-lipofuscinosis, neuronal 6 | 1,417930806 |
| 1450717_at | Ang | angiogenin, ribonuclease, RNase A family, 5 | 1,41784055 |
| 1424885_at | Klhdc8a | kelch domain containing 8A | 1,417056278 |
| 1426208_x_at | Plagl1 | pleiomorphic adenoma gene-like 1 | 1,416403156 |
| 1421098_at | Stap1 | signal transducing adaptor family member 1 | 1,415588713 |
| 1426546_at | Tesk2 | testis-specific kinase 2 | 1,415172619 |
| 1437367_at | Bat1a | HLA-B-associated transcript 1A | 1,413248366 |
| 1424649_a_at | Tspan8 | tetraspanin 8 | 1,41314342 |
| 1438165_x_at | Vat1 | vesicle amine transport protein 1 homolog (T californica) | 1,412025011 |
| 1424401_at | Aldh1l1 | aldehyde dehydrogenase 1 family, member L1 | 1,411486764 |
| 1431833_a_at | Hmgcs2 | 3-hydroxy-3-methylglutaryl-Coenzyme A synthase 2 | 1,411284296 |
| 1460190_at | Ap1m2 | adaptor protein complex AP-1, mu 2 subunit | 1,411027059 |
| 1421628_at | Il18r1 | interleukin 18 receptor 1 | 1,410855518 |
| 1418724_at | Cfi | complement component factor i | 1,410006368 |
| 1449843_at | St8sia2 | ST8 alpha-N-acetyl-neuraminide alpha-2,8-sialyltransferase 2 | 1,409842643 |
| 1421350_a_at | Grip1 | glutamate receptor interacting protein 1 | 1,408532048 |
| 1417893_at | Sfxn3 | sideroflexin 3 | 1,407829625 |
| 1419006_s_at | Peli2 | pellino 2 | 1,40744611 |
| 1455694_at | Nbeal2 | neurobeachin-like 2 | 1,407030301 |
| 1418213_at | Krt23 | keratin 23 | 1,402761973 |
| 1417741_at | Pygl | liver glycogen phosphorylase | 1,402561633 |
| 1417695_a_at | Soat1 | sterol O-acyltransferase 1 | 1,398636432 |
| 1421376_at | Traf6 | TNF receptor-associated factor 6 | 1,395612173 |
| 1456395_at | Ppargc1a | peroxisome proliferative activated receptor, gamma, coactivator 1 alpha | 1,395097632 |
| 1426472_at | Zfp52 | zinc finger protein 52 | 1,394907277 |
| 1452203_at | Obfc2a | oligonucleotide/oligosaccharide-binding fold containing 2A | 1,394124888 |
| 1434959_at | Dhh | desert hedgehog | 1,391728979 |
| 1451489_at | Slc25a35 | solute carrier family 25, member 35 | 1,391563004 |
| 1418856_a_at | Fanca | Fanconi anemia, complementation group A | 1,391502754 |
| 1453486_a_at | Scube2 | signal peptide, CUB domain, EGF-like 2 | 1,390063013 |
| 1419447_s_at | Tbc1d1 | TBC1 domain family, member 1 | 1,389973168 |
| 1449877_s_at | Kifc1 | kinesin family member C1 | 1,389648198 |
| 1417476_at | Fbxw5 | F-box and WD-40 domain protein 5 | 1,389366482 |
| 1437013_x_at | Atp6v0b | ATPase, H+ transporting, lysosomal V0 subunit B | 1,388470987 |
| 1422184_a_at | Ak1 | adenylate kinase 1 | 1,388359526 |
| 1426568_at | Slc2a9 | solute carrier family 2 (facilitated glucose transporter), member 9 | 1,388023158 |
| 1423877_at | Chaf1b | chromatin assembly factor 1, subunit B (p60) | 1,387021591 |
| 1456225_x_at | Trib3 | tribbles homolog 3 (Drosophila) | 1,386676996 |
| 1423443_at | Slc4a8 | solute carrier family 4 (anion exchanger), member 8 | 1,386541895 |
| 1456251_x_at | Tspo | translocator protein | 1,385690947 |
| 1419814_s_at | S100a1 | S100 calcium binding protein A1 | 1,385619322 |
| 1449112_at | Slc27a5 | solute carrier family 27 (fatty acid transporter), member 5 | 1,384833635 |
| 1416184_s_at | Hmga1 /// Hmga1-rs1 | high mobility group AT-hook 1 /// high mobility group AT-hook I, related sequence 1 | 1,384811969 |
| 1451828_a_at | Acsl4 | acyl-CoA synthetase long-chain family member 4 | 1,383194781 |
| 1451431_a_at | Dbndd2 | dysbindin (dystrobrevin binding protein 1) domain containing 2 | 1,382247385 |
| 1437746_at | Lrrtm1 | leucine rich repeat transmembrane neuronal 1 | 1,381969514 |
| 1452109_at | Il17re | interleukin 17 receptor E | 1,381086245 |
| 1417810_a_at | Kcnb1 /// Pacsin2 | potassium voltage gated channel, Shab-related subfamily, member 1 /// protein kinase C and casein kinase substrate in neurons 2 | 1,380110819 |
| 1418032_at | Itfg2 | integrin alpha FG-GAP repeat containing 2 | 1,379034196 |
| 1419113_at | Ap1g2 | adaptor protein complex AP-1, gamma 2 subunit | 1,378295132 |
| 1415945_at | Mcm5 | minichromosome maintenance deficient 5, cell division cycle 46 (S. cerevisiae) | 1,37743397 |
| 1419125_at | Ptpn18 | protein tyrosine phosphatase, non-receptor type 18 | 1,37606249 |
| AFFX-r2-Bs-lys-5_at | --- | --- | 1,375371893 |
| 1426730_a_at | Prl2b1 | prolactin family 2, subfamily b, member 1 | 1,374090935 |
| 1448698_at | Ccnd1 | cyclin D1 | 1,373044114 |
| 1424475_at | Camkk2 | calcium/calmodulin-dependent protein kinase kinase 2, beta | 1,372248181 |
| 1426227_s_at | Vps37c | vacuolar protein sorting 37C (yeast) | 1,372015006 |
| 1418903_at | Aqp2 | aquaporin 2 | 1,371581624 |
| 1426454_at | Arhgdib | Rho, GDP dissociation inhibitor (GDI) beta | 1,370962902 |
| 1457849_at | --- | --- | 1,370643228 |
| 1416823_a_at | Osbpl1a | oxysterol binding protein-like 1A | 1,370469678 |
| 1439479_at | Lct | lactase | 1,370142859 |
| 1451021_a_at | Klf5 | Kruppel-like factor 5 | 1,369992287 |
| 1424833_at | Itpr2 | inositol 1,4,5-triphosphate receptor 2 | 1,368114711 |
| 1455490_at | Pigr | polymeric immunoglobulin receptor | 1,367644731 |
| 1420795_at | Fgf9 | fibroblast growth factor 9 | 1,365978228 |
| 1428283_at | Cyp2s1 | cytochrome P450, family 2, subfamily s, polypeptide 1 | 1,365483697 |
| 1434137_x_at | Zg16 | zymogen granule protein 16 | 1,364983221 |
| 1438571_at | Bub1 | budding uninhibited by benzimidazoles 1 homolog (S. cerevisiae) | 1,364575615 |
| 1418746_at | Pnkd | paroxysmal nonkinesiogenic dyskinesia | 1,364179208 |
| 1450264_a_at | Chka | choline kinase alpha | 1,363799075 |
| 1429240_at | Stard4 | StAR-related lipid transfer (START) domain containing 4 | 1,362540299 |
| 1417308_at | Pkm2 | pyruvate kinase, muscle | 1,362488163 |
| 1456222_at | Btbd1 | BTB (POZ) domain containing 1 | 1,36228666 |
| 1437974_a_at | Hk1 | hexokinase 1 | 1,362250437 |
| 1424363_at | Mycbpap | MYCBP associated protein | 1,360936274 |
| 1415903_at | Slc38a1 | solute carrier family 38, member 1 | 1,359868025 |
| 1451887_at | Lrba | LPS-responsive beige-like anchor | 1,358971225 |
| 1434272_at | Cpeb2 | cytoplasmic polyadenylation element binding protein 2 | 1,358666824 |
| 1448886_at | Gata3 | GATA binding protein 3 | 1,358534898 |
| 1435221_at | Foxp1 | forkhead box P1 | 1,358206387 |
| 1422666_at | Cblc | Casitas B-lineage lymphoma c | 1,357483904 |
| 1424776_a_at | Slc25a28 | solute carrier family 25, member 28 | 1,357328729 |
| 1435222_at | Foxp1 | forkhead box P1 | 1,357232707 |
| 1419490_at | Fam19a5 | family with sequence similarity 19, member A5 | 1,356967937 |
| 1428357_at | 2610019F03Rik | RIKEN cDNA 2610019F03 gene | 1,356010262 |
| 1423124_x_at | Rad54l | RAD54 like (S. cerevisiae) | 1,355890918 |
| 1424864_at | Hipk2 | homeodomain interacting protein kinase 2 | 1,355820869 |
| 1426102_at | Cyp2j13 | cytochrome P450, family 2, subfamily j, polypeptide 13 | 1,355192771 |
| 1449151_at | Cdk18 | cyclin-dependent kinase 18 | 1,354650711 |
| 1429128_x_at | Nfkb2 | nuclear factor of kappa light polypeptide gene enhancer in B-cells 2, p49/p100 | 1,354635734 |
| 1449049_at | Tlr1 | toll-like receptor 1 | 1,354072588 |
| 1425302_at | Mfsd6l | major facilitator superfamily domain containing 6-like | 1,352639042 |
| 1450131_a_at | Bspry | B-box and SPRY domain containing | 1,352073045 |
| 1449732_at | Zscan21 | zinc finger and SCAN domain containing 21 | 1,350752984 |
| 1455531_at | Mfsd4 | major facilitator superfamily domain containing 4 | 1,348160982 |
| 1427633_a_at | Pappa | pregnancy-associated plasma protein A | 1,347920355 |
| 1426237_at | Sp2 | Sp2 transcription factor | 1,346543021 |
| 1460243_at | Sptlc2 | serine palmitoyltransferase, long chain base subunit 2 | 1,346353134 |
| 1427363_at | --- | --- | 1,345527442 |
| 1415842_at | Mlst8 | MTOR associated protein, LST8 homolog (S. cerevisiae) | 1,345041205 |
| 1418294_at | Epb4.1l4b | erythrocyte protein band 4.1-like 4b | 1,344918422 |
| 1419126_at | Hoxd9 | homeobox D9 | 1,344870631 |
| 1425753_a_at | Ung | uracil DNA glycosylase | 1,343263195 |
| 1421075_s_at | Cyp7b1 | cytochrome P450, family 7, subfamily b, polypeptide 1 | 1,342500284 |
| 1416528_at | Sh3bgrl3 | SH3 domain binding glutamic acid-rich protein-like 3 | 1,340480467 |
| 1426284_at | Krt20 | keratin 20 | 1,340409555 |
| 1451750_at | Irak4 | interleukin-1 receptor-associated kinase 4 | 1,339972246 |
| 1460336_at | Ppargc1a | peroxisome proliferative activated receptor, gamma, coactivator 1 alpha | 1,339759905 |
| 1419619_at | Krt80 | keratin 80 | 1,339674378 |
| 1435358_at | Cuedc1 | CUE domain containing 1 | 1,338676574 |
| 1439567_at | Tbx3 | T-box 3 | 1,338439334 |
| 1434048_at | 4930471M23Rik | RIKEN cDNA 4930471M23 gene | 1,338336761 |
| 1417518_at | Plagl2 | pleiomorphic adenoma gene-like 2 | 1,338235253 |
| 1418743_a_at | Tesc | tescalcin | 1,338101863 |
| 1422804_at | Serpinb6b | serine (or cysteine) peptidase inhibitor, clade B, member 6b | 1,337902503 |
| 1421933_at | Cbx5 | chromobox homolog 5 (Drosophila HP1a) | 1,337169767 |
| 1436833_x_at | Ttll1 | tubulin tyrosine ligase-like 1 | 1,336584228 |
| 1425098_at | Zfp106 | zinc finger protein 106 | 1,336202481 |
| 1425900_at | Hkdc1 | hexokinase domain containing 1 | 1,335214766 |
| 1460417_at | AB041803 | cDNA sequence AB041803 | 1,335184626 |
| AFFX-LysX-M_at | --- | --- | 1,33500153 |
| 1421140_a_at | Foxp1 | forkhead box P1 | 1,334142694 |
| 1454897_at | 6330509M05Rik | RIKEN cDNA 6330509M05 gene | 1,333906 |
| 1418711_at | Pdgfa | platelet derived growth factor, alpha | 1,332103287 |
| 1425411_at | Arl4a | ADP-ribosylation factor-like 4A | 1,33165003 |
| 1448408_at | Hps1 | Hermansky-Pudlak syndrome 1 homolog (human) | 1,331547007 |
| 1423276_at | Ildr1 | immunoglobulin-like domain containing receptor 1 | 1,331443277 |
| 1455338_at | A4galt | alpha 1,4-galactosyltransferase | 1,331218383 |
| 1438956_x_at | Pim3 | proviral integration site 3 | 1,330046621 |
| 1417050_at | C1qtnf4 | C1q and tumor necrosis factor related protein 4 | 1,329721049 |
| AFFX-PheX-5_at | --- | --- | 1,329679316 |
| 1451752_at | Foxk1 | forkhead box K1 | 1,328739938 |
| 1438173_x_at | Pmf1 | polyamine-modulated factor 1 | 1,328580614 |
| 1451461_a_at | Aldoc | aldolase C, fructose-bisphosphate | 1,327413402 |
| 1423123_at | Rad54l | RAD54 like (S. cerevisiae) | 1,327133704 |
| 1418917_at | Hebp2 | heme binding protein 2 | 1,326344445 |
| 1417568_at | Ncald | neurocalcin delta | 1,325549283 |
| 1423509_a_at | Iapp | islet amyloid polypeptide | 1,325513393 |
| 1424143_a_at | Cdt1 | chromatin licensing and DNA replication factor 1 | 1,325426144 |
| 1419851_at | Slc4a8 | solute carrier family 4 (anion exchanger), member 8 | 1,325407902 |
| AFFX-18SRNAMur/X00686_M_at | --- | --- | 1,323551857 |
| 1425159_at | Golt1a | golgi transport 1 homolog A (S. cerevisiae) | 1,323499995 |
| 1425919_at | Ndufa12 | NADH dehydrogenase (ubiquinone) 1 alpha subcomplex, 12 | 1,323144552 |
| AFFX-r2-Bs-lys-3_at | --- | --- | 1,322697798 |
| 1434138_at | Prune | prune homolog (Drosophila) | 1,322524761 |
| 1417829_a_at | Rab15 | RAB15, member RAS oncogene family | 1,320711934 |
| 1426028_a_at | Cit | citron | 1,319173171 |
| 1434752_at | LOC100044751 | hypothetical LOC100044751 | 1,319078298 |
| 1425470_at | --- | --- | 1,318441337 |
| AFFX-LysX-3_at | --- | --- | 1,317892894 |
| 1460365_a_at | Dnm1 | dynamin 1 | 1,317886204 |
| 1426996_at | Fam83h | family with sequence similarity 83, member H | 1,317873781 |
| 1417396_at | Podxl | podocalyxin-like | 1,317773136 |
| 1425263_a_at | Mbp | myelin basic protein | 1,31759784 |
| 1423726_at | Vat1 | vesicle amine transport protein 1 homolog (T californica) | 1,316401805 |
| AFFX-LysX-5_at | --- | --- | 1,316099251 |
| 1418485_at | Slc4a3 | solute carrier family 4 (anion exchanger), member 3 | 1,316037599 |
| 1420059_at | Fam54b | Family with sequence similarity 54, member B | 1,315896722 |
| 1451139_at | Slc39a4 | solute carrier family 39 (zinc transporter), member 4 | 1,315569984 |
| 1434325_x_at | Prkar1b | protein kinase, cAMP dependent regulatory, type I beta | 1,315376445 |
| 1417470_at | Apobec3 | apolipoprotein B mRNA editing enzyme, catalytic polypeptide 3 | 1,315209661 |
| 1420096_at | Zscan21 | Zinc finger and SCAN domain containing 21 | 1,31456822 |
| 1426808_at | Lgals3 | lectin, galactose binding, soluble 3 | 1,314264356 |
| 1456585_x_at | E130309D02Rik | RIKEN cDNA E130309D02 gene | 1,313561699 |
| 1449061_a_at | Prim1 | DNA primase, p49 subunit | 1,313299146 |
| 1451687_a_at | Hnf1b | HNF1 homeobox B | 1,313220884 |
| 1420553_x_at | Serpina1a | serine (or cysteine) peptidase inhibitor, clade A, member 1A | 1,31228283 |
| 1427057_at | Nt5dc3 | 5-nucleotidase domain containing 3 | 1,312165924 |
| 1451132_at | Pbxip1 | pre-B-cell leukemia transcription factor interacting protein 1 | 1,311870018 |
| 1438948_x_at | Tspo | translocator protein | 1,311788295 |
| 1417297_at | Itpr3 | inositol 1,4,5-triphosphate receptor 3 | 1,311470178 |
| 1452416_at | Il6ra | interleukin 6 receptor, alpha | 1,309606212 |
| 1456606_a_at | Chst11 /// Phactr1 | carbohydrate sulfotransferase 11 /// phosphatase and actin regulator 1 | 1,309488897 |
| 1437325_x_at | Aldh18a1 | aldehyde dehydrogenase 18 family, member A1 | 1,309269795 |
| 1419551_s_at | Stk39 | serine/threonine kinase 39, STE20/SPS1 homolog (yeast) | 1,308550234 |
| 1433881_at | Dnajc11 | DnaJ (Hsp40) homolog, subfamily C, member 11 | 1,30854202 |
| 1435105_at | Rnf208 | ring finger protein 208 | 1,308078862 |
| 1448850_a_at | Dnajc5 | DnaJ (Hsp40) homolog, subfamily C, member 5 | 1,308009296 |
| 1417389_at | Gpc1 | glypican 1 | 1,307788961 |
| 1452292_at | Ap2b1 | adaptor-related protein complex 2, beta 1 subunit | 1,307369413 |
| 1417038_at | Sept9 | septin 9 | 1,307222518 |
| 1449638_at | C920021L13Rik | RIKEN cDNA C920021L13 gene | 1,30710359 |
| AFFX-r2-Bs-phe-M_at | --- | --- | 1,304063409 |
| 1433775_at | C77080 | expressed sequence C77080 | 1,303704858 |
| 1435458_at | Pim1 | proviral integration site 1 | 1,302430734 |
| 1417300_at | Smpdl3b | sphingomyelin phosphodiesterase, acid-like 3B | 1,301031734 |
| 1449109_at | Socs2 | suppressor of cytokine signaling 2 | 1,300611713 |
| 1419103_a_at | Abhd6 | abhydrolase domain containing 6 | 1,299901644 |
| 1423122_at | Avpi1 | arginine vasopressin-induced 1 | 1,298762599 |
| 1424879_at | Lrch4 /// Lrch4-sap25 /// Sap25 | leucine-rich repeats and calponin homology (CH) domain containing 4 /// Lrch4-Sap25 gene /// sin3 associated polypeptide | 1,29868705 |
| 1451596_a_at | Sphk1 | sphingosine kinase 1 | 1,2986426 |
| 1419916_at | Rnf20 | Ring finger protein 20 | 1,297912721 |
| 1429124_s_at | Rexo1 | REX1, RNA exonuclease 1 homolog (S. cerevisiae) | 1,297231478 |
| 1420576_at | Lpar2 | lysophosphatidic acid receptor 2 | 1,296746654 |
| 1436886_x_at | Xab2 | XPA binding protein 2 | 1,296350536 |
| 1421223_a_at | Anxa4 | annexin A4 | 1,295520089 |
| 1460569_x_at | Cldn3 | claudin 3 | 1,295429606 |
| 1448028_at | Tbc1d24 | TBC1 domain family, member 24 | 1,29410456 |
| 1452730_at | Rps4y2 | ribosomal protein S4, Y-linked 2 | 1,293609487 |
| 1420575_at | Mt3 | metallothionein 3 | 1,293481001 |
| 1428740_a_at | Pigt | phosphatidylinositol glycan anchor biosynthesis, class T | 1,293053514 |
| 1415958_at | Slc2a4 | solute carrier family 2 (facilitated glucose transporter), member 4 | 1,293000264 |
| 1421504_at | Sp4 | trans-acting transcription factor 4 | 1,291690742 |
| 1449953_at | Itgb3bp | integrin beta 3 binding protein (beta3-endonexin) | 1,291683515 |
| 1421482_at | Bsnd | Bartter syndrome, infantile, with sensorineural deafness (Barttin) | 1,291507621 |
| 1427178_at | Tmc4 | transmembrane channel-like gene family 4 | 1,291342505 |
| 1416627_at | Spint1 | serine protease inhibitor, Kunitz type 1 | 1,291034764 |
| 1433918_at | Atg4d | autophagy-related 4D (yeast) | 1,288229955 |
| AFFX-PheX-M_at | --- | --- | 1,288214564 |
| 1449511_a_at | Ssbp4 | single stranded DNA binding protein 4 | 1,287941877 |
| 1452273_at | Fam83h | family with sequence similarity 83, member H | 1,286687296 |
| 1449982_at | Il11 | interleukin 11 | 1,286519854 |
| 1427287_s_at | Itpr2 | inositol 1,4,5-triphosphate receptor 2 | 1,286404669 |
| 1416689_at | Tuft1 | tuftelin 1 | 1,286275344 |
| 1452426_x_at | --- | --- | 1,285407087 |
| 1424304_at | Tpcn2 | two pore segment channel 2 | 1,2852005 |
| 1460739_at | Zmiz2 | zinc finger, MIZ-type containing 2 | 1,2849951 |
| 1424814_a_at | Bcl2l14 | BCL2-like 14 (apoptosis facilitator) | 1,284913863 |
| 1424441_at | Slc27a4 | solute carrier family 27 (fatty acid transporter), member 4 | 1,284756928 |
| AFFX-ThrX-3_at | --- | --- | 1,284405988 |
| 1419977_s_at | Esrp2 | epithelial splicing regulatory protein 2 | 1,284339638 |
| 1425362_at | Agfg2 | ArfGAP with FG repeats 2 | 1,283959073 |
| 1439436_x_at | Incenp | inner centromere protein | 1,283930157 |
| 1450127_a_at | Gcgr | glucagon receptor | 1,282825546 |
| 1417629_at | Prodh | proline dehydrogenase | 1,282167917 |
| 1460344_at | Pbxip1 | pre-B-cell leukemia transcription factor interacting protein 1 | 1,281822068 |
| 1424850_at | Map3k1 | mitogen-activated protein kinase kinase kinase 1 | 1,281802425 |
| 1419835_s_at | Plec | plectin | 1,281697044 |
| 1420160_s_at | Myo1e | myosin IE | 1,28111864 |
| 1435077_at | Asxl1 | additional sex combs like 1 (Drosophila) | 1,281000518 |
| 1456037_x_at | Preb | prolactin regulatory element binding | 1,280684901 |
| 1419601_at | Kcnj10 | potassium inwardly-rectifying channel, subfamily J, member 10 | 1,280201444 |
| 1416854_at | Slc34a2 | solute carrier family 34 (sodium phosphate), member 2 | 1,279624994 |
| 1434155_a_at | 2310061I04Rik | RIKEN cDNA 2310061I04 gene | 1,279352041 |
| 1455678_at | Sema4b | sema domain, immunoglobulin domain (Ig), transmembrane domain (TM) and short cytoplasmic domain, (semaphorin) 4B | 1,278745356 |
| 1424351_at | Wfdc2 | WAP four-disulfide core domain 2 | 1,278429135 |
| 1417640_at | Cd79b | CD79B antigen | 1,278355299 |
| 1416802_a_at | Cdca5 | cell division cycle associated 5 | 1,27791245 |
| 1450955_s_at | Sort1 | sortilin 1 | 1,277839105 |
| 1418555_x_at | Spic | Spi-C transcription factor (Spi-1/PU.1 related) | 1,277486984 |
| 1448817_at | Otub1 | OTU domain, ubiquitin aldehyde binding 1 | 1,277382221 |
| 1433485_x_at | Gpr56 | G protein-coupled receptor 56 | 1,277334409 |
| 1418611_at | Gpr162 | G protein-coupled receptor 162 | 1,27706776 |
| 1451553_at | Art5 | ADP-ribosyltransferase 5 | 1,276987631 |
| 1417822_at | D17H6S56E-5 | DNA segment, Chr 17, human D6S56E 5 | 1,276972133 |
| 1438317_a_at | Endog | endonuclease G | 1,275642091 |
| 1460746_at | Fignl1 | fidgetin-like 1 | 1,275468932 |
| 1425685_at | Akr1b10 | Aldo-keto reductase family 1, member B10 (aldose reductase) | 1,275002979 |
| 1419068_at | Rabgef1 | RAB guanine nucleotide exchange factor (GEF) 1 | 1,274989978 |
| 1439269_x_at | Mcm7 | minichromosome maintenance deficient 7 (S. cerevisiae) | 1,274780052 |
| 1421142_s_at | Foxp1 | forkhead box P1 | 1,27461114 |
| 1453851_a_at | Gadd45g | growth arrest and DNA-damage-inducible 45 gamma | 1,274453608 |
| 1424590_at | Ddx19b | DEAD (Asp-Glu-Ala-Asp) box polypeptide 19b | 1,274231247 |
| 1418706_at | Slc38a3 | solute carrier family 38, member 3 | 1,273963679 |
| 1451224_at | Scamp5 | secretory carrier membrane protein 5 | 1,273935443 |
| 1452233_at | Abcc1 | ATP-binding cassette, sub-family C (CFTR/MRP), member 1 | 1,273782854 |
| 1428788_at | Pgp | phosphoglycolate phosphatase | 1,273767073 |
| 1417801_a_at | Ppfibp2 | PTPRF interacting protein, binding protein 2 (liprin beta 2) | 1,272977834 |
| 1436810_x_at | 2900010M23Rik | RIKEN cDNA 2900010M23 gene | 1,272947678 |
| 1418719_at | Haus8 | 4HAUS augmin-like complex, subunit 8 | 1,2727853 |
| 1424834_s_at | Itpr2 | inositol 1,4,5-triphosphate receptor 2 | 1,272360955 |
| 1419217_at | Sergef | secretion regulating guanine nucleotide exchange factor | 1,272304297 |
| 1420295_x_at | Clcn5 | chloride channel 5 | 1,27194628 |
| 1448950_at | Il1r1 | interleukin 1 receptor, type I | 1,271552068 |
| 1450333_a_at | Gata2 | GATA binding protein 2 | 1,271409157 |
| 1422150_at | Hmx3 | H6 homeobox 3 | 1,271339038 |
| 1418671_at | Capn5 | calpain 5 | 1,270850675 |
| 1425745_a_at | Tacc2 | transforming, acidic coiled-coil containing protein 2 | 1,269946946 |
| 1448595_a_at | Bex1 | brain expressed gene 1 | 1,269053062 |
| 1417738_at | Rab25 | RAB25, member RAS oncogene family | 1,268295687 |
| 1459890_s_at | 1110008P14Rik | RIKEN cDNA 1110008P14 gene | 1,268081531 |
| 1419550_a_at | Stk39 | serine/threonine kinase 39, STE20/SPS1 homolog (yeast) | 1,267983334 |
| 1417065_at | Egr1 | early growth response 1 | 1,267770114 |
| 1424421_at | Flad1 | RFad1, flavin adenine dinucleotide synthetase, homolog (yeast) | 1,267274364 |
| 1448146_at | Wwp2 | WW domain containing E3 ubiquitin protein ligase 2 | 1,267139266 |
| 1416780_at | Pfkm | phosphofructokinase, muscle | 1,266844206 |
| 1449519_at | Gadd45a | growth arrest and DNA-damage-inducible 45 alpha | 1,266808327 |
| 1417228_at | Capn1 | calpain 1 | 1,266301691 |
| 1424577_at | Msto1 | misato homolog 1 (Drosophila) | 1,266225481 |
| 1428794_at | Specc1 | sperm antigen with calponin homology and coiled-coil domains 1 | 1,265594939 |
| 1434853_x_at | Mkrn1 | makorin, ring finger protein, 1 | 1,265522144 |
| 1424612_at | Nipal2 | NIPA-like domain containing 2 | 1,265089344 |
| 1420012_at | Xbp1 | X-box binding protein 1 | 1,265028189 |
| 1435114_at | Wdhd1 | WD repeat and HMG-box DNA binding protein 1 | 1,26473163 |
| 1439081_at | Mgea5 | meningioma expressed antigen 5 (hyaluronidase) | 1,26370888 |
| 1425741_at | Srgap3 | SLIT-ROBO Rho GTPase activating protein 3 | 1,263151633 |
| 1416387_at | Pip4k2c | phosphatidylinositol-5-phosphate 4-kinase, type II, gamma | 1,263024563 |
| 1436487_x_at | Fbxw2 | F-box and WD-40 domain protein 2 | 1,262750602 |
| 1433797_at | E130309D02Rik | RIKEN cDNA E130309D02 gene | 1,262733687 |
| 1437724_x_at | Pitpnm1 | phosphatidylinositol transfer protein, membrane-associated 1 | 1,262377316 |
| 1427749_at | Galnt3 | UDP-N-acetyl-alpha-D-galactosamine:polypeptide N-acetylgalactosaminyltransferase 3 | 1,262302203 |
| 1422735_at | Foxq1 | forkhead box Q1 | 1,261576284 |
| 1417420_at | Ccnd1 | cyclin D1 | 1,261103169 |
| 1422220_at | Pou1f1 | POU domain, class 1, transcription factor 1 | 1,260981358 |
| 1451229_at | Hdac11 | histone deacetylase 11 | 1,260770365 |
| 1423733_a_at | Fiz1 | Flt3 interacting zinc finger protein 1 | 1,260077903 |
| 1429400_at | Clcn5 | chloride channel 5 | 1,259976469 |
| 1434976_x_at | Eif4ebp1 | eukaryotic translation initiation factor 4E binding protein 1 | 1,259770189 |
| 1418577_at | Trim8 | tripartite motif-containing 8 | 1,259706426 |
| 1423150_at | Scg5 | secretogranin V | 1,259434641 |
| 1419154_at | Tmprss2 | transmembrane protease, serine 2 | 1,259250276 |
| 1435655_at | Rpl12 | ribosomal protein L12 | 1,259199199 |
| 1451083_s_at | Aars | alanyl-tRNA synthetase | 1,259173941 |
| 1433808_at | Heatr7a | HEAT repeat containing 7A | 1,259152712 |
| 1420028_s_at | Mcm3 | minichromosome maintenance deficient 3 (S. cerevisiae) | 1,258918267 |
| 1452465_at | Myh1 | myosin, heavy polypeptide 1, skeletal muscle, adult | 1,258483459 |
| 1426652_at | Mcm3 | minichromosome maintenance deficient 3 (S. cerevisiae) | 1,257844287 |
| 1451303_at | BC002230 | cDNA sequence BC002230 | 1,257843616 |
| 1455131_at | Opa3 | optic atrophy 3 (human) | 1,257632807 |
| 1455019_x_at | Ckap4 | cytoskeleton-associated protein 4 | 1,257265897 |
| 1424817_at | Spef1 | sperm flagellar 1 | 1,256968303 |
| 1418840_at | Pdcd4 | programmed cell death 4 | 1,256866072 |
| 1417616_at | St6galnac2 | ST6 (alpha-N-acetyl-neuraminyl-2,3-beta-galactosyl-1,3)-N-acetylgalactosaminide alpha-2,6-sialyltransferase 2 | 1,256856107 |
| 1424327_at | 3200002M19Rik | RIKEN cDNA 3200002M19 gene | 1,25669567 |
| 1452226_at | Rcc2 | regulator of chromosome condensation 2 | 1,256600426 |
| 1427397_at | 2810046L04Rik | RIKEN cDNA 2810046L04 gene | 1,256564611 |
| 1451814_a_at | Htatip2 | HIV-1 tat interactive protein 2, homolog (human) | 1,256492014 |
| 1448810_at | Gne | glucosamine | 1,256260566 |
| 1417971_at | Nrm | nurim (nuclear envelope membrane protein) | 1,256155583 |
| 1424022_at | Osgin1 | oxidative stress induced growth inhibitor 1 | 1,256133948 |
| 1437046_x_at | Fam63a | family with sequence similarity 63, member A | 1,255946981 |
| 1425553_s_at | Hip1r | huntingtin interacting protein 1 related | 1,255914281 |
| 1424174_at | Shkbp1 | Sh3kbp1 binding protein 1 | 1,25588231 |
| 1424119_at | Prkab1 | protein kinase, AMP-activated, beta 1 non-catalytic subunit | 1,254338871 |
| 1454759_at | Git1 | G protein-coupled receptor kinase-interactor 1 | 1,254138748 |
| 1453571_at | Depdc6 | DEP domain containing 6 | 1,253989863 |
| 1460635_at | Fastk | Fas-activated serine/threonine kinase | 1,253535797 |
| 1434813_x_at | Wars | tryptophanyl-tRNA synthetase | 1,253418319 |
| 1416110_at | Slc35a4 | solute carrier family 35, member A4 | 1,253314233 |
| 1423431_a_at | Mybbp1a | MYB binding protein (P160) 1a | 1,253213466 |
| 1416852_a_at | Ncdn | neurochondrin | 1,252919528 |
| 1419132_at | Tlr2 | toll-like receptor 2 | 1,252909296 |
| 1424214_at | Parm1 | prostate androgen-regulated mucin-like protein 1 | 1,252220414 |
| 1435951_at | Grip1 | glutamate receptor interacting protein 1 | 1,251712293 |
| 1426754_x_at | Ckap4 | cytoskeleton-associated protein 4 | 1,250837111 |
| 1416449_x_at | Stxbp2 | syntaxin binding protein 2 | 1,250819682 |
| 1447984_at | D1Ertd75e | DNA segment, Chr 1, ERATO Doi 75, expressed | 1,250189447 |
| 1456515_s_at | Tcfl5 | transcription factor-like 5 (basic helix-loop-helix) | 1,249671981 |
| 1419134_at | Rhbg | Rhesus blood group-associated B glycoprotein | 1,249550032 |
| 1437615_s_at | Vps37c | vacuolar protein sorting 37C (yeast) | 1,249465981 |
| 1425072_at | Skp2 | S-phase kinase-associated protein 2 (p45) | 1,248923645 |
| 1429086_at | Gm16136 /// Grhl2 /// LOC100503499 | predicted gene 16136 /// grainyhead-like 2 (Drosophila) /// hypothetical LOC100503499 | 1,248903178 |
| 1450985_a_at | Tjp2 | tight junction protein 2 | 1,248796268 |
| 1434651_a_at | Cldn3 | claudin 3 | 1,248531056 |
| 1415952_at | Mark2 | MAP/microtubule affinity-regulating kinase 2 | 1,248332335 |
| 1451166_a_at | Ccdc101 | coiled-coil domain containing 101 | 1,247949982 |
| 1423707_at | Tmem50b | transmembrane protein 50B | 1,24776873 |
| 1455026_at | Sbno1 | sno, strawberry notch homolog 1 (Drosophila) | 1,247749291 |
| 1433869_at | Zxdc | ZXD family zinc finger C | 1,247707665 |
| 1422511_a_at | Ogfr | opioid growth factor receptor | 1,247594253 |
| 1448232_x_at | Gm5620 /// Gm6682 /// Tuba1a /// Tuba1b /// Tuba1c | predicted gene 5620 /// predicted gene 6682 /// tubulin, alpha 1A /// tubulin, alpha 1B /// tubulin, alpha 1C | 1,24744569 |
| 1451803_a_at | Vegfb | vascular endothelial growth factor B | 1,246949298 |
| AFFX-r2-Bs-thr-3_s_at | --- | --- | 1,246747969 |
| 1415873_a_at | Actr1a | ARP1 actin-related protein 1 homolog A, centractin alpha (yeast) | 1,245950854 |
| 1424359_at | Oplah | 5-oxoprolinase (ATP-hydrolysing) | 1,245792239 |
| 1439375_x_at | Aldoa | aldolase A, fructose-bisphosphate | 1,245703798 |
| AFFX-r2-Bs-phe-3_at | --- | --- | 1,245257818 |
| 1419195_at | Wfdc15b | WAP four-disulfide core domain 15B | 1,244927235 |
| 1451168_a_at | Arhgdia | Rho GDP dissociation inhibitor (GDI) alpha | 1,243358908 |
| 1436404_at | Tlcd1 | TLC domain containing 1 | 1,243329388 |
| 1455788_x_at | Poldip3 | polymerase (DNA-directed), delta interacting protein 3 | 1,243054718 |
| 1435122_x_at | Dnmt1 | DNA methyltransferase (cytosine-5) 1 | 1,242868437 |
| 1450057_at | Sys1 | SYS1 Golgi-localized integral membrane protein homolog (S. cerevisiae) | 1,242160455 |
| 1459892_at | Psg19 | pregnancy specific glycoprotein 19 | 1,2419744 |
| 1450862_at | Rad54l | RAD54 like (S. cerevisiae) | 1,241556184 |
| 1434859_at | Umps | uridine monophosphate synthetase | 1,241538606 |
| 1423257_at | Cyp4a14 | cytochrome P450, family 4, subfamily a, polypeptide 14 | 1,241121139 |
| 1436615_a_at | Otc | ornithine transcarbamylase | 1,241070513 |
| 1417431_a_at | Sphk2 | sphingosine kinase 2 | 1,240685079 |
| 1451555_at | Nln | neurolysin (metallopeptidase M3 family) | 1,24005443 |
| 1418507_s_at | Socs2 | suppressor of cytokine signaling 2 | 1,239956801 |
| 1427997_at | Ndufaf4 | NADH dehydrogenase (ubiquinone) 1 alpha subcomplex, assembly factor 4 | 1,239669869 |
| 1436454_x_at | Fen1 | flap structure specific endonuclease 1 | 1,239666263 |
| 1453752_at | Rpl17 | ribosomal protein L17 | 1,239358454 |
| AFFX-r2-Bs-phe-5_at | --- | --- | 1,23920787 |
| 1424456_at | Pvrl2 | poliovirus receptor-related 2 | 1,238397502 |
| 1448354_at | G6pdx | glucose-6-phosphate dehydrogenase X-linked | 1,238392342 |
| 1449406_at | Cyhr1 | cysteine and histidine rich 1 | 1,238315005 |
| 1416125_at | Fkbp5 | FK506 binding protein 5 | 1,23810515 |
| 1438321_x_at | Fam63a | family with sequence similarity 63, member A | 1,237451419 |
| 1418831_at | Pkp3 | plakophilin 3 | 1,237433067 |
| AFFX-r2-Bs-dap-3_at | --- | --- | 1,237045533 |
| 1451183_at | Myo19 | myosin XIX | 1,236861174 |
| 1416305_at | Sh3bp4 | SH3-domain binding protein 4 | 1,236723879 |
| 1424383_at | Tmem51 | transmembrane protein 51 | 1,23670726 |
| 1422603_at | Rnase4 | ribonuclease, RNase A family 4 | 1,236500499 |
| 1417774_at | Nans | N-acetylneuraminic acid synthase (sialic acid synthase) | 1,235686153 |
| 1460672_at | 2410002F23Rik | RIKEN cDNA 2410002F23 gene | 1,235601857 |
| 1416069_at | Pfkp | phosphofructokinase, platelet | 1,235362659 |
| 1422545_at | Tbx2 | T-box 2 | 1,235095783 |
| 1417591_at | Ptges2 | prostaglandin E synthase 2 | 1,234887113 |
| 1449337_at | Tdo2 | tryptophan 2,3-dioxygenase | 1,234828446 |
| 1421406_at | --- | --- | 1,234067645 |
| 1448495_at | Tsta3 | tissue specific transplantation antigen P35B | 1,234021049 |
| 1425163_at | AI661453 | expressed sequence AI661453 | 1,233828605 |
| 1448868_at | Scand1 | SCAN domain-containing 1 | 1,232722399 |
| 1433702_at | Ermp1 | endoplasmic reticulum metallopeptidase 1 | 1,232565414 |
| 1448740_at | Rangrf | RAN guanine nucleotide release factor | 1,232336011 |
| 1424400_a_at | Aldh1l1 /// LOC100047937 | aldehyde dehydrogenase 1 family, member L1 /// 10-formyltetrahydrofolate dehydrogenase-like | 1,231500072 |
| 1455350_at | Tmem62 | transmembrane protein 62 | 1,231368616 |
| 1450628_at | Slc2a8 | solute carrier family 2, (facilitated glucose transporter), member 8 | 1,230865236 |
| 1449187_at | Pdgfa | platelet derived growth factor, alpha | 1,229751004 |
| 1437666_x_at | Ubc | ubiquitin C | 1,229608868 |
| 1416896_at | Rps6ka1 | ribosomal protein S6 kinase polypeptide 1 | 1,229451942 |
| 1460223_a_at | Epb4.9 | erythrocyte protein band 4.9 | 1,22896403 |
| 1426822_at | Rhot2 | ras homolog gene family, member T2 | 1,228720436 |
| 1431653_at | Tcrb-J | T-cell receptor beta, joining region | 1,228633742 |
| 1418488_s_at | Ripk4 | receptor-interacting serine-threonine kinase 4 | 1,228601688 |
| 1434034_at | Cerk | ceramide kinase | 1,228564733 |
| 1421425_a_at | Rcan2 | regulator of calcineurin 2 | 1,228198481 |
| 1421019_at | 1700021F05Rik | RIKEN cDNA 1700021F05 gene | 1,228115473 |
| 1448685_at | 2900010M23Rik | RIKEN cDNA 2900010M23 gene | 1,228108671 |
| 1429005_at | Mfhas1 | malignant fibrous histiocytoma amplified sequence 1 | 1,227797634 |
| 1455958_s_at | Pptc7 | PTC7 protein phosphatase homolog (S. cerevisiae) | 1,227781504 |
| 1422824_s_at | Eps8 | epidermal growth factor receptor pathway substrate 8 | 1,227414027 |
| 1456005_a_at | Bcl2l11 | BCL2-like 11 (apoptosis facilitator) | 1,226989472 |
| 1450650_at | Myo10 | myosin X | 1,22652119 |
| 1437531_at | Trpm1 | transient receptor potential cation channel, subfamily M, member 1 | 1,225485102 |
| 1418301_at | Irf6 | interferon regulatory factor 6 | 1,224970485 |
| 1416641_at | Lig1 | ligase I, DNA, ATP-dependent | 1,224636954 |
| 1416419_s_at | Gabarapl1 | gamma-aminobutyric acid (GABA) A receptor-associated protein-like 1 | 1,223744159 |
| 1460700_at | Stat3 | signal transducer and activator of transcription 3 | 1,222875844 |
| 1424432_at | Ubtd1 | ubiquitin domain containing 1 | 1,222708485 |
| 1421134_at | Areg | amphiregulin | 1,221605795 |
| 1455959_s_at | Gclc | glutamate-cysteine ligase, catalytic subunit | 1,221205541 |
| 1418477_at | Matn1 | matrilin 1, cartilage matrix protein | 1,220988489 |
| 1437751_at | Ppargc1a | peroxisome proliferative activated receptor, gamma, coactivator 1 alpha | 1,220780176 |
| 1448169_at | Krt18 | keratin 18 | 1,220772086 |
| 1448574_at | Nme6 | non-metastatic cells 6, protein expressed in (nucleoside-diphosphate kinase) | 1,220693666 |
| 1449052_a_at | Dnmt3b | DNA methyltransferase 3B | 1,220382138 |
| 1426655_a_at | Fam63a | family with sequence similarity 63, member A | 1,220346197 |
| 1420196_s_at | Tbc1d14 | TBC1 domain family, member 14 | 1,220063925 |
| 1423362_at | Sort1 | sortilin 1 | 1,219899151 |
| 1426966_at | Axin1 | axin 1 | 1,21985179 |
| 1417654_at | Sdc4 | syndecan 4 | 1,219362768 |
| 1426750_at | Flnb | filamin, beta | 1,218707235 |
| 1455447_at | D430019H16Rik | RIKEN cDNA D430019H16 gene | 1,218163544 |
| 1415763_a_at | 2510006D16Rik /// LOC100505293 | RIKEN cDNA 2510006D16 gene /// hypothetical LOC100505293 | 1,217821961 |
| 1426898_at | Tab1 | TGF-beta activated kinase 1/MAP3K7 binding protein 1 | 1,217280695 |
| 1424385_at | Gon4l | gon-4-like (C.elegans) | 1,216851546 |
| 1453207_at | 2900053A13Rik /// LOC100503890 | RIKEN cDNA 2900053A13 gene /// hypothetical protein LOC100503890 | 1,216794565 |
| 1416328_a_at | Atp6v0e | ATPase, H+ transporting, lysosomal V0 subunit E | 1,21673676 |
| 1418186_at | Gstt1 | glutathione S-transferase, theta 1 | 1,216536532 |
| 1418521_a_at | Mtx1 | metaxin 1 | 1,215578521 |
| 1426209_at | Strn4 | striatin, calmodulin binding protein 4 | 1,215290064 |
| 1436549_a_at | Hnrnpa1 | heterogeneous nuclear ribonucleoprotein A1 | 1,214122374 |
| 1425284_a_at | Rab27a | RAB27A, member RAS oncogene family | 1,214113413 |
| 1452005_at | Dlat | dihydrolipoamide S-acetyltransferase (E2 component of pyruvate dehydrogenase complex) | 1,214080516 |
| 1451399_at | Pnkd | paroxysmal nonkinesiogenic dyskinesia | 1,213505451 |
| 1423646_at | Zdhhc3 | zinc finger, DHHC domain containing 3 | 1,213326491 |
| 1422050_at | Nkx1-2 | NK1 transcription factor related, locus 2 (Drosophila) | 1,212140449 |
| 1432827_x_at | Ubc | ubiquitin C | 1,212084611 |
| 1460693_a_at | Col9a3 | collagen, type IX, alpha 3 | 1,211918079 |
| 1460198_a_at | Psmb3 | proteasome (prosome, macropain) subunit, beta type 3 | 1,211737817 |
| 1455539_at | Gm9983 | predicted gene 9983 | 1,21167875 |
| 1417346_at | Pycard | PYD and CARD domain containing | 1,210728784 |
| 1452919_a_at | Pgp | phosphoglycolate phosphatase | 1,210683523 |
| 1424463_at | Mfsd6 | major facilitator superfamily domain containing 6 | 1,210599969 |
| 1438320_s_at | Mcm7 | minichromosome maintenance deficient 7 (S. cerevisiae) | 1,210395105 |
| 1423686_a_at | Prr13 | proline rich 13 | 1,210360143 |
| 1455579_at | Csn1s2a | casein alpha s2-like A | 1,210145708 |
| 1424514_at | Rnf126 | ring finger protein 126 | 1,208687583 |
| 1437454_a_at | Tmx2 | thioredoxin-related transmembrane protein 2 | 1,208614281 |
| 1438151_x_at | Zdhhc14 | zinc finger, DHHC domain containing 14 | 1,208259142 |
| 1455927_x_at | Nsmce1 | Non-SMC element 1 homolog (S. cerevisiae) | 1,206905793 |
| 1448866_at | Senp3 | SUMO/sentrin specific peptidase 3 | 1,206872072 |
| 1436959_x_at | Nelf | nasal embryonic LHRH factor | 1,206453166 |
| 1454802_x_at | Arih2 | ariadne homolog 2 (Drosophila) | 1,20642104 |
| 1460169_a_at | Cdk16 | cyclin-dependent kinase 16 | 1,206115875 |
| 1420520_x_at | Eral1 | Era (G-protein)-like 1 (E. coli) | 1,205289442 |
| 1452359_at | Rell1 | RELT-like 1 | 1,204221739 |
| 1424159_at | Fam134c | family with sequence similarity 134, member C | 1,203222439 |
| 1460409_at | Cpt1a | carnitine palmitoyltransferase 1a, liver | 1,202606299 |
| AFFX-DapX-3_at | --- | --- | 1,201513678 |
| 1451703_s_at | Aprt | adenine phosphoribosyl transferase | 1,201334292 |
| 1426248_at | Stk24 | serine/threonine kinase 24 (STE20 homolog, yeast) | 1,201280728 |
| 1416031_s_at | Mcm7 | minichromosome maintenance deficient 7 (S. cerevisiae) | 1,201204858 |
| 1452055_at | Ctdsp1 | CTD (carboxy-terminal domain, RNA polymerase II, polypeptide A) small phosphatase 1 | 1,201109431 |
| 1428327_at | Trak1 | trafficking protein, kinesin binding 1 | 1,200841914 |
| 1424572_a_at | H2afy | H2A histone family, member Y | 1,200148815 |
| 1425552_at | Hip1r | huntingtin interacting protein 1 related | 1,199811182 |
| 1426818_at | Arrdc4 | arrestin domain containing 4 | 1,199515392 |
| 1454979_at | Diap1 | diaphanous homolog 1 (Drosophila) | 1,199484521 |
| 1451357_at | Mpnd | MPN domain containing | 1,199341024 |
| 1448393_at | Cldn7 | claudin 7 | 1,199280149 |
| 1455826_a_at | Bace1 | beta-site APP cleaving enzyme 1 | 1,199228073 |
| 1434543_a_at | Bola2 | bolA-like 2 (E. coli) | 1,196954961 |
| 1427243_at | Rell1 | RELT-like 1 | 1,196931075 |
| AFFX-r2-Bs-dap-M_at | --- | --- | 1,196659874 |
| 1424052_at | Thap4 | THAP domain containing 4 | 1,196409475 |
| 1421529_a_at | Txnrd1 | thioredoxin reductase 1 | 1,195517483 |
| 1416606_s_at | Nhp2 | NHP2 ribonucleoprotein homolog (yeast) | 1,195410455 |
| 1426465_at | Dlgap4 | discs, large homolog-associated protein 4 (Drosophila) | 1,195337845 |
| 1436542_at | --- | --- | 1,195316115 |
| 1425927_a_at | Atf5 | activating transcription factor 5 | 1,195255627 |
| 1420494_x_at | Ubc | ubiquitin C | 1,194993362 |
| 1448154_at | Ndrg2 | N-myc downstream regulated gene 2 | 1,193338279 |
| 1415755_a_at | Ube2v1 | ubiquitin-conjugating enzyme E2 variant 1 | 1,193211683 |
| 1460690_at | Fam195b | family with sequence similarity 195, member B | 1,192424737 |
| 1450887_at | Rqcd1 | rcd1 (required for cell differentiation) homolog 1 (S. pombe) | 1,192402245 |
| 1460561_x_at | Sepw1 | selenoprotein W, muscle 1 | 1,192073714 |
| 1455955_s_at | Snx17 | sorting nexin 17 | 1,191921529 |
| 1437043_a_at | Fam125a | family with sequence similarity 125, member A | 1,191768176 |
| 1423679_at | 2810432L12Rik | RIKEN cDNA 2810432L12 gene | 1,190799647 |
| AFFX-r2-Ec-bioC-5_at | --- | --- | 1,190768875 |
| 1423685_at | Aars | alanyl-tRNA synthetase | 1,190145018 |
| 1437343_x_at | Atad3a | ATPase family, AAA domain containing 3A | 1,189835897 |
| 1424929_a_at | Trim26 | tripartite motif-containing 26 | 1,189644802 |
| 1437503_a_at | Shisa5 | shisa homolog 5 (Xenopus laevis) | 1,189533812 |
| 1426064_at | Cyp3a44 | cytochrome P450, family 3, subfamily a, polypeptide 44 | 1,189263574 |
| 1453572_a_at | Plp2 | proteolipid protein 2 | 1,187227128 |
| 1433948_at | Pla2g1b | Phospholipase A2, group IB, pancreas | 1,187064324 |
| 1437297_at | Chd8 | chromodomain helicase DNA binding protein 8 | 1,185904267 |
| 1418908_at | Pam | peptidylglycine alpha-amidating monooxygenase | 1,185851775 |
| 1417713_at | Eif2s2 | eukaryotic translation initiation factor 2, subunit 2 (beta) | 1,183178083 |
| 1415918_a_at | Tpi1 | triosephosphate isomerase 1 | 1,182521293 |
| 1416608_a_at | BC004004 | cDNA sequence BC004004 | 1,181197246 |
| 1436715_s_at | Cdipt | CDP-diacylglycerol--inositol 3-phosphatidyltransferase (phosphatidylinositol synthase) | 1,180279553 |
| 1417068_a_at | Ptpn1 | protein tyrosine phosphatase, non-receptor type 1 | 1,180052298 |
| 1420997_a_at | Gpi1 | glucose phosphate isomerase 1 | 1,178323557 |
| 1435416_x_at | Pigq | phosphatidylinositol glycan anchor biosynthesis, class Q | 1,178216875 |
| 1419803_s_at | Ccdc12 | coiled-coil domain containing 12 | 1,178205058 |
| 1419273_at | C80913 | expressed sequence C80913 | 1,178069562 |
| 1424262_at | Aif1l | allograft inflammatory factor 1-like | 1,177057877 |
| 1423626_at | Dst | dystonin | 1,176946444 |
| 1417433_at | Lypla2 | lysophospholipase 2 | 1,176715878 |
| 1435431_at | Psmg4 | proteasome (prosome, macropain) assembly chaperone 4 | 1,174481077 |
| 1416945_at | Ptov1 | prostate tumor over expressed gene 1 | 1,173600126 |
| 1424296_at | Gclc | glutamate-cysteine ligase, catalytic subunit | 1,173528485 |
| 1449635_at | Prpf19 | PRP19/PSO4 pre-mRNA processing factor 19 homolog (S. cerevisiae) | 1,172635254 |
| 1450081_x_at | Gpi1 | glucose phosphate isomerase 1 | 1,172204684 |
| 1423202_a_at | Ncor1 | nuclear receptor co-repressor 1 | 1,172203367 |
| 1438357_at | Pfdn5 | prefoldin 5 | 1,172198157 |
| 1419839_x_at | Prpf19 | PRP19/PSO4 pre-mRNA processing factor 19 homolog (S. cerevisiae) | 1,169017231 |
| 1450809_at | Sval2 | seminal vesicle antigen-like 2 | 1,167737604 |
| 1439405_x_at | Cntd1 | Cyclin N-terminal domain containing 1 | 1,164178455 |
| 1423702_at | H1f0 | H1 histone family, member 0 | 1,162273008 |
| 1455713_x_at | Phb2 | prohibitin 2 | 1,158253923 |
| 1434814_x_at | Gpi1 | glucose phosphate isomerase 1 | 1,155699256 |
| 1422506_a_at | Cstb | cystatin B | 1,154942614 |
| 1438925_x_at | Atp6v0c | ATPase, H+ transporting, lysosomal V0 subunit C | 1,153853045 |
| 1418091_at | Tcfcp2l1 | transcription factor CP2-like 1 | 1,150684073 |
| 1456580_s_at | Atp5d | ATP synthase, H+ transporting, mitochondrial F1 complex, delta subunit | 1,150492604 |
| 1419491_at | Defb1 | defensin beta 1 | 1,137775462 |
| 1455319_x_at | Rps8 | ribosomal protein S8 | 1,132807061 |
| 1456306_a_at | Umod | uromodulin | 1,130708182 |
| 1448245_at | Rpsa | ribosomal protein SA | 1,101284085 |
|  |  |  |  |
